# Resolution, conflict and rate shifts: insights from a densely sampled plastome phylogeny for *Rhododendron* (Ericaceae)

**DOI:** 10.1093/aob/mcac114

**Published:** 2022-09-10

**Authors:** Zhi-Qiong Mo, Chao-Nan Fu, Ming-Shu Zhu, Richard I Milne, Jun-Bo Yang, Jie Cai, Han-Tao Qin, Wei Zheng, Peter M Hollingsworth, De-Zhu Li, Lian-Ming Gao

**Affiliations:** CAS Key Laboratory for Plant Diversity and Biogeography of East Asia, Kunming Institute of Botany, Chinese Academy of Sciences, Kunming 650201, Yunnan, China; Germplasm Bank of Wild Species, Kunming Institute of Botany, Chinese Academy of Sciences, Kunming 650201, Yunnan, China; University of the Chinese Academy of Sciences, Beijing 100049, China; CAS Key Laboratory for Plant Diversity and Biogeography of East Asia, Kunming Institute of Botany, Chinese Academy of Sciences, Kunming 650201, Yunnan, China; Germplasm Bank of Wild Species, Kunming Institute of Botany, Chinese Academy of Sciences, Kunming 650201, Yunnan, China; CAS Key Laboratory for Plant Diversity and Biogeography of East Asia, Kunming Institute of Botany, Chinese Academy of Sciences, Kunming 650201, Yunnan, China; University of the Chinese Academy of Sciences, Beijing 100049, China; Institute of Molecular Plant Sciences, School of Biological Sciences, University of Edinburgh, Edinburgh EH9 3JH, UK; Germplasm Bank of Wild Species, Kunming Institute of Botany, Chinese Academy of Sciences, Kunming 650201, Yunnan, China; Germplasm Bank of Wild Species, Kunming Institute of Botany, Chinese Academy of Sciences, Kunming 650201, Yunnan, China; CAS Key Laboratory for Plant Diversity and Biogeography of East Asia, Kunming Institute of Botany, Chinese Academy of Sciences, Kunming 650201, Yunnan, China; University of the Chinese Academy of Sciences, Beijing 100049, China; CAS Key Laboratory for Plant Diversity and Biogeography of East Asia, Kunming Institute of Botany, Chinese Academy of Sciences, Kunming 650201, Yunnan, China; University of the Chinese Academy of Sciences, Beijing 100049, China; Royal Botanic Garden Edinburgh, Edinburgh EH3 5LR, UK; CAS Key Laboratory for Plant Diversity and Biogeography of East Asia, Kunming Institute of Botany, Chinese Academy of Sciences, Kunming 650201, Yunnan, China; Germplasm Bank of Wild Species, Kunming Institute of Botany, Chinese Academy of Sciences, Kunming 650201, Yunnan, China; University of the Chinese Academy of Sciences, Beijing 100049, China; CAS Key Laboratory for Plant Diversity and Biogeography of East Asia, Kunming Institute of Botany, Chinese Academy of Sciences, Kunming 650201, Yunnan, China; Lijiang Forest Biodiversity National Observation and Research Station, Kunming Institute of Botany, Chinese Academy of Sciences, Lijiang 674100, Yunnan, China

**Keywords:** Himalaya–Hengduan Mountains, *Rhododendron*, genome skimming, plastid genome, phylogenomics, diversification, recent radiation

## Abstract

**Background and Aims:**

*Rhododendron* is a species-rich and taxonomically challenging genus due to recent adaptive radiation and frequent hybridization. A well-resolved phylogenetic tree would help to understand the diverse history of *Rhododendron* in the Himalaya–Hengduan Mountains where the genus is most diverse.

**Methods:**

We reconstructed the phylogeny based on plastid genomes with broad taxon sampling, covering 161 species representing all eight subgenera and all 12 sections, including ~45 % of the *Rhododendron* species native to the Himalaya–Hengduan Mountains. We compared this phylogeny with nuclear phylogenies to elucidate reticulate evolutionary events and clarify relationships at all levels within the genus. We also estimated the timing and diversification history of *Rhododendron*, especially the two species-rich subgenera *Rhododendron* and *Hymenanthes* that comprise >90 % of *Rhododendron* species in the Himalaya–Hengduan Mountains.

**Key Results:**

The full plastid dataset produced a well-resolved and supported phylogeny of *Rhododendron*. We identified 13 clades that were almost always monophyletic across all published phylogenies. The conflicts between nuclear and plastid phylogenies suggested strongly that reticulation events may have occurred in the deep lineage history of the genus. Within *Rhododendron*, subgenus *Therorhodion* diverged first at 56 Mya, then a burst of diversification occurred from 23.8 to 17.6 Mya, generating ten lineages among the component 12 clades of core *Rhododendron*. Diversification in subgenus *Rhododendron* accelerated *c*. 16.6 Mya and then became fairly continuous. Conversely, *Hymenanthes* diversification was slow at first, then accelerated very rapidly around 5 Mya. In the Himalaya–Hengduan Mountains, subgenus *Rhododendron* contained one major clade adapted to high altitudes and another to low altitudes, whereas most clades in *Hymenanthes* contained both low- and high-altitude species, indicating greater ecological plasticity during its diversification.

**Conclusions:**

The 13 clades proposed here may help to identify specific ancient hybridization events. This study will help to establish a stable and reliable taxonomic framework for *Rhododendron*, and provides insight into what drove its diversification and ecological adaption. Denser sampling of taxa, examining both organelle and nuclear genomes, is needed to better understand the divergence and diversification history of *Rhododendron*.

## INTRODUCTION

A robust phylogeny is essential to understand the process of spatiotemporal evolution of a plant group (Jansen *et al.*, 2007; Olofsson *et al.*, 2019). For a species-rich group, intensive sampling of both genomes and taxa is necessary to recover a robust phylogeny (Barrett *et al.*, 2013; Li *et al.*, 2019), especially for groups where hybridization is common and gene trees may not correspond to species trees (Kong *et al.*, 2021; Li *et al.*, 2022). Until recently, resource limitation and costs forced most studies to choose between heavy sampling of either genomes or taxa, but the increasing accessibility and affordability of next-generation sequencing (NGS) data now permits both (Barrett *et al.*, 2013; H. T. Li *et al.*, 2021). Hence the phylogenomic method may be employed, using a large amount of genetic data from chloroplast, mitochondrial and nuclear genomes (Steele and Pires, 2011; Yu *et al.*, 2018), using approaches such as genome skimming, transcriptome and target enrichment sequencing (e.g. Zeng *et al.*, 2017; Villaverde *et al.*, 2018; H. T. Li *et al.*, 2021). Among these methods, genome skimming is now commonly used to cost-effectively and efficiently obtain the plastid genome. The plastid genome has numerous advantages for phylogenetic reconstruction, including uniparental inheritance, minimal recombination, and conservation of structure and evolutionary rate, but with sufficient characters for phylogenetic inference (Petit and Vendramin, 2007). Therefore, the plastid genome has been successfully used for molecular systematics at various taxonomic levels among angiosperms (Straub *et al.*, 2012; Gitzendanner *et al.*, 2018; Zhang *et al.*, 2020; H. T. Li *et al.*, 2021), notably within exceptionally species-rich genera such as *Acacia* (Williams *et al.*, 2016) and *Begonia* (Li *et al.*, 2022).

*Rhododendron*, a species-rich and taxonomically challenging genus in Ericaceae, comprises more than 1000 species (Chamberlain *et al.*, 1996), making it the largest genus of woody plants in the Northern Hemisphere (Wu *et al.*, 2003). *Rhododendron* is among the world’s most horticulturally valuable genera (Craven, 2011), but is also a vital component of montane ecosystems (Gibbs *et al.*, 2011; Kumar, 2012), containing many dominant or ecologically important species that contribute to the stability of alpine or subalpine plant communities (Wu *et al.*, 2003). One section (*Vireya* = *Schistanthe*) has radiated explosively in Southeast Asia, mainly in the Malay Peninsula, New Guinea and the islands between (Brown *et al.*, 2006; Goetsch *et al.*, 2011), whereas two subgenera (*Hymenanthes* and *Rhododendron*) have both diversified greatly in the Himalaya–Hengduan Mountains, together generating >90 % of the region’s >320 *Rhododendron* species, among which about two-thirds are endemic (Chamberlain *et al.*, 1996; Fang *et al.*, 2005; Yan *et al.*, 2015; Fu *et al.*, 2022). Diversification of *Rhododendron* species in the Himalaya–Hengduan Mountains was associated with uplifts of the Tibetan plateau and climate change during the Neogene (Shrestha *et al.*, 2018; Ding *et al.*, 2020; Xia *et al.*, 2022).

A well-resolved phylogenetic tree would be of help to understand the diverse history of *Rhododendron* in the Himalaya–Hengduan Mountains, and shed light on the geological history of this area, as well as to the classification, conservation and utilization of this genus. However, rapid radiation into large numbers of species tends to generate very short phylogeny branches, hampering accurate phylogenetic resolution. Furthermore, resolution in many previous phylogenetic studies of *Rhododendron* has been hampered by insufficient sampling and/or the use of only a few DNA loci (Kurashige *et al.*, 1998, 2001; Gao *et al.*, 2002; Goetsch *et al.*, 2005; Milne *et al.*, 2010; Berry *et al.*, 2018; Shrestha *et al.*, 2018). Moreover, conflict among these studies probably reflects reticulate evolution, which might in turn have contributed to poor resolution of some key nodes. Numerous *Rhododendron* species occur sympatrically, and considerable interspecific hybridization/introgression events occur among them (Milne *et al.*, 1999, 2003; Zhang *et al.*, 2007; Milne and Abbott, 2008; Ma *et al.*, 2010; Yan *et al.*, 2017; Zheng *et al.*, 2021). There are hence two particular challenges to generating a well-resolved and accurate phylogeny for *Rhododendron*: recent and rapid speciation, and reticulate evolution, both of which raise a challenge for phylogenetic inference and species identification in *Rhododendron*, whether based on morphology or molecular data (e.g. Yan *et al.*, 2015; Fu *et al.*, 2022).

There is agreement that subgenus *Therorhodion* is the first diverging group within *Rhododendron* (Gao *et al.*, 2002; Goetsch *et al.*, 2005; Xia *et al.*, 2022), but the major divergence events that followed have tended to be neither well resolved nor agreed between studies. Xia *et al.* (2022) resolved deep phylogenetic relationships with strong support using 3437 orthologous nuclear genes from transcriptome data, but some species relationships had weak support, and there was conflict with their own plastid data, which were derived from 38 plastid protein-coding genes via transcriptome data. This cytonuclear discordance included both deep clade relationships and species relationships (e.g. within *Hymenanthes*), and there was also considerable missing plastid data, especially for key species such as *R*. *semibarbatum* and *R*. *canadense* (Xia *et al.*, 2022). Only by combining a highly resolved plastid phylogeny with a nuclear one can the evolution of this genus be properly understood, because neither alone is likely to represent the species tree. Hence in the current study, near-complete plastid genomes of 161 sampled species were recovered using genome skimming data to reconstruct the plastid phylogeny, representing all 12 sections of eight subgenera recognized in *Rhododendron* (Chamberlain *et al.*, 1996). Correct species identification is crucial for phylogenetic inference, but challenging within *Rhododendron*, so almost all species examined here were confirmed based on previous DNA barcoding studies (Yan *et al.*, 2015; Fu *et al.*, 2022). We used this phylogeny to estimate the timing and history of diversification in *Rhododendron*, especially in the Himalaya–Hengduan Mountains. In addition, we compared our plastid phylogeny with published nuclear phylogenies (especially Xia *et al.*, 2022), to elucidate reticulate evolution events and clarify relationships at all levels within the genus, as well as to provide a resource for further research.

## MATERIALS AND METHODS

### Taxon sampling

A total of 161 species representing all eight subgenera (*Hymenanthes*, *Rhododendron*, *Tsutsusi*, *Pentanthera*, *Azaleastrum*, *Therorhodion*, *Mumeazalea* and *Candidastrum*) and 12 sections (*Ponticum*, *Pogonanthum*, *Rhododendron*, *Vireya*, *Brachycalyx*, *Tsutsusi*, *Pentanthera*, *Rhodora*, *Sciadorhodion*, *Viscidula*, *Azaleastrum* and *Choniastrum*) of *Rhododendron* recognized by Chamberlain *et al.* (1996) as well as the main lineages in other studies (Goetsch *et al.*, 2005; Xia *et al.*, 2022) were included in this study. Plastomes of 138 species from four subgenera, *Hymenanthes*, *Rhododendron*, *Tsutsusi* and *Azaleastrum*, were obtained from our previous study (Fu *et al.*, 2022), and to these were added newly generated plastomes of 23 *Rhododendron* species from the other four subgenera, from genome skimming data, making 161 in total. Of these, 142 species occur in the Himalaya–Hengduan Mountains. Three species, *Erica glandulosa*, *Diplarche multiflora* and *Empetrum nigrum*, were selected as outgroups. Healthy and fresh leaves were collected and dried immediately in silica gel. Most vouchers were deposited at the Herbarium of Kunming Institute of Botany (KUN), Chinese Academy of Sciences. Detailed information of sampling, classification, vouchers and sources of data is provided in Supplementary Data Table S1.

### DNA extraction, sequencing, assembly and annotation

Total genomic DNA was extracted from silica-gel-dried leaves using a modified CTAB method (Doyle and Doyle, 1987). Total DNA was quantified and sheared to a mean insert size of 500 bp for Illumina library construction following standard protocols (NEBNext^®^ Ultra IITMDNA Library Prep Kit for Illumina^®^). The libraries were sequenced to generate ~2 Gb data for each species on the Illumina HiSeq X Ten platform (Illumina, San Diego, CA, USA) with 150-bp paired-end reads at BGI Wuhan, China.

Plastomes of the newly sampled species were *de novo* assembled from genome skimming data using the GetOrganelle toolkit (Jin *et al.*, 2020). In this toolkit, target-associated plastomic reads were recruited via Bowtie2 v2.3.4 (Langmead and Salzberg, 2012), extracted from total genomic reads, and subsequently *de novo* assembled by SPAdes v3.15 (Bankevich *et al.*, 2012). As previously described (Fu *et al.*, 2022), it is extremely difficult to obtain the complete plastid genome of *Rhododendron* and the outgroups in Ericaceae from genome skimming data. Therefore, the plastid genome scaffolds were annotated and checked as implemented in Geneious v9.0.2 (Kearse *et al.*, 2012) using as a reference the published plastome of *Rhododendron delavayi* (GenBank accession: NC_047438), which was assembled using Illumina and PacBio sequencing data.

### Sequence alignment, substitution saturation and selective pressure analyses

The protein-coding genes, rRNA genes and non-coding regions (the last referring to both introns and intergenic regions between protein-coding genes and/or rRNA genes, throughout this paper) were separately extracted from the annotated plastid genome scaffolds using the Python script get_annotated_regions_from_gb.py (available from https://github.com/Kinggerm/PersonalUtilities/). Multiple sequence alignment for each locus was performed using MAFFT v7.471 (Katoh and Standley, 2013), the alignments were manually modified in Geneious, and the protein-coding genes were aligned using the ‘translation align’ option.

Substitutional saturation was assessed for each protein-coding gene in DAMBE v7.0.68 (Xia, 2018) and measured using the substitution saturation index (Iss). From this no substitution saturation was detected, so all protein-coding genes obtained here were included for subsequent analyses. Furthermore, the CodeML program implemented in PAML v4.9h (Yang, 2007) was used to estimate the ratio Ka/Ks (i.e. ω) of the non-synonymous substitution rate (Ka) to synonymous substitution rate (Ks) for each protein-coding gene.

### Phylogenetic dataset construction and analysis

We obtained 72 protein-coding genes, 63 non-coding regions and four rRNA genes in total. By concatenating sequences in different combinations, three supermatrices (datasets) were formed: WP contained all 139 loci, NCS comprised the 63 non-coding regions, and PCS comprised 72 protein-coding genes plus four rRNA genes. To investigate the phylogenetic effect of genes under positive selection, two additional datasets, WP-ω and PCS-ω, were formed, by removing those genes under positive selection from the WP and PCS datasets, respectively.

Maximum likelihood (ML) and Bayesian inference (BI) methods were performed based on the WP dataset. ML analysis was conducted with a GTR + Γ substitution model and 1000 rapid bootstrap replicates, using RAxML v8.2.12 (Stamatakis, 2006). In addition, an ML tree of the WP dataset was also constructed using IQ-TREE v1.6.10 (Nguyen *et al.*, 2015) under the MFP option with 1000 ultrafast bootstrap (UFBS) replicates (Hoang *et al.*, 2018). For the BI method, two independent tree searches of PhyloBayes MPI analysis starting from a random tree were run until the likelihood of the sampled trees had stabilized and converged (maxdiff < 0.3), with constant sites removed (-dc), and trees and associated model parameters sampled every cycle under the CAT + GTR + Γ (four discrete gamma rates) substitution model, using PhyloBayes MPI v1.8c (Lartillot *et al.*, 2013). ML analyses were also performed based on the NCS, PCS, WP-ω and PCS-ω datasets using RAxML and IQ-TREE respectively under the same parameters as before. Trees were visualized in FigTree v1.4.3 (available from http://tree.bio.ed.ac.uk/software/figtree/).

### Divergence time estimation

To compare the divergence time estimated by different approaches, three methods (Bayesian, RelTime and penalized likelihood) were used in divergence time estimates. Divergence time was estimated using the full plastid dataset (WP) with the Bayesian approach conducted in BEAST v1.8.4 (Drummond *et al.*, 2012). BEAST analysis was run under a relaxed molecular clock with uncorrelated, lognormally distributed substitution rates for each branch in the phylogenetic tree, the GTR + Γ + I nucleotide substitution model and a birth–death incomplete sampling speciation process tree prior. The dated tree was calibrated with two fossils. The leaf fossil of *Rhododendron protodilatatum* (Tanai and Onoe, 1961; Ozaki, 1980) dated to the start of the Pliocene [*c*. 5.3 million years ago (Mya)] was set as the minimum age constraint of the crown of sect. *Brachycalyx* [priors for the time to the most recent common ancestor (tMRCA): lognormal distribution with mean = 6, lognormal SD = 1 and offset = 5.3]. The seed fossil of *R. newburyanum* (Collinson and Crane, 1978) dated to the late Paleocene (*c*. 56 Mya) was set as the minimum age constraint of the *Rhododendron* crown group (priors for tMRCA: mean = 61, lognormal SD = 4 and offset = 56). All other priors were set to their default values. Two independent Markov chain Monte Carlo (MCMC) runs that were started with a random starting tree and sampled every 50 000 generations were conducted with the same parameters for a total of 2 × 10^9^ generations. Stationarity and convergence were assessed using Tracer v1.7.1 (Rambaut *et al.*, 2018), and Effective Sample Sizes (ESS) of all parameters exceeding 200 were considered convergent. The initial 25 % of trees sampled in each run were discarded as burn-in, and the remaining trees were combined into a single file using LogCombiner v1.8.4, and TreeAnnotator v1.8.4 (Drummond *et al.*, 2012) was used to find the maximum clade credibility (MCC) tree, which was finally visualized using FigTree.

Penalized likelihood and RelTime (Tamura *et al.*, 2012) approaches were also used to estimate divergence times for WP in treePL v1.0 (Smith and O’Meara, 2012) and MEGA X (Kumar *et al.*, 2018), respectively. The same two fossil calibration points as for BEAST were used in both cases. For treePL analysis, 1000 ML bootstrap trees with branch length generated by RAxML were used as the input trees. A priming analysis was first performed to determine the best optimization parameter values, followed by a cross-validation analysis to determine the optimal smoothing parameter value. The RelTime method was performed based on the ML tree from the WP dataset, which which was built by RAxML, with the parameters set following Xia *et al.* (2022).

### Diversification analyses

To test whether the choice of method would influence the results, three approaches (BAMM, LTT and MEDUSA) were used to estimate the diversification dynamics within *Rhododendron*. The outgroup taxa were discarded and only the species of *Rhododendron* were retained from the MCC tree generated by BEAST analysis. We utilized BAMM v2.5.0 (Rabosky, 2014) to assess the historical diversification rate change over time of *Rhododendron*. First, the setBAMMpriors function in BAMMtools v2.1.7 (Rabosky, 2014) was used to generate prior parameters for the ultrametric phylogenetic tree. If the calibrated chronogram was not fully sampled and only contained part of the species diversity of the genus, it may lead to biased estimates of diversification rates on molecular phylogenies (FitzJohn *et al.*, 2009; Rabosky, 2014). Therefore, we performed BAMM analyses with a sampling fraction file to correct non-random incomplete taxon sampling. Species of *Menziesia* were treated as members of sect. *Sciadorhodion*, as proposed by Craven (2011). In the fraction file, tips (i.e. sampled species) were assigned to groups following a thorough survey, and group assignment was conducted as follows. First, all taxonomically recognized subgroups (Chamberlain *et al.*, 1996) that were resolved as monophyletic had the species number of that group assigned, at the lowest possible taxonomic level, i.e. subsection or section where possible. However, within the subg. *Hymenanthes*, not all subsections were monophyletic, so species numbers had to be applied at the subgenus level. The same was true in the two major clades of subg. *Rhododendron* that did not contain the three basal groups (subsections *Micrantha*, *Ledum* and sect. *Vireya*), so the species number was set according to the total species number of all the section(s)/subsection(s) contained in each clade. In cases where sections were not monophyletic (e.g. *Sciadorhodion* and *Rhodora*), constituent clades were identified, and the taxonomic literature was used to estimate species numbers for each clade. The MCMC chain was then run for 2 × 10^7^ generations and sampled every 10 000 generations in BAMM. Finally, BAMMtools was used to summarize rates over each branch and plot diversification rates over time from the output data of BAMM. The convergence (ESS > 200) was assessed, with the first 15 % of samples discarded as burn-in using the R package coda v0.19 (Plummer *et al.*, 2006). With the expected number of shifts set to a prior value of 1, the single best shift configuration with the maximum a posteriori (MAP) probability was found for generating the phylorate plot. In addition, a rate-through-time (net diversification, speciation and extinction rates) curve was plotted using the plotRateThroughTime function. The divergence age and species diversification rate of the two major subgenera (*Rhododendron* and *Hymenanthes*) that are diverse in the Himalaya–Hengduan Mountains were extracted from the results of BEAST and BAMM respectively, and averages were taken to compare their diversification rate and species age (i.e. the time when a species diverged from its nearest sampled relative). The analyses were repeated with groups that occur entirely outside the Himalaya–Hengduan Mountains excluded, i.e. sect. *Vireya* and subsect. *Ledum* from subg. *Rhododendron*, and subsect. *Pontica* from *Hymenanthes*, to allow direct comparisons of diversification rates and species ages within the region.

The semi-logarithmic lineage through time (LTT) plot was drawn by APE v5.5 (Paradis and Schliep, 2019) to estimate the overall diversification pattern. A total of 2000 trees were randomly selected from the BEAST analysis to calculate the confidence intervals.

The diversification rate across the phylogeny of *Rhododendron* was also inferred, once again based on the MCC tree, using the R package MEDUSA v0.955 (Alfaro *et al.*, 2009) applying default settings [i.e. the corrected Akaike information criterion (AICc) and mixed mode]. The species richness of each monophyletic group was consistent with the assignments of BAMM, and the MCC tree was pruned to contain the assigned groups so that each terminal reflected a monophyletic group. The species richness was assigned to each terminal branch.

## RESULTS

### Characteristics of the datasets

All *Rhododendron* species sampled here failed to provide a complete circular structure, but sequencing data could be assembled into many long plastome scaffolds. From these, annotation and extraction was achieved for 72 of the 75–78 protein-coding genes present in the *Rhododendron* plastome, plus 63 non-coding regions and four rRNA genes, ensuring that missing data for each species was <25 % (Supplementary Data Table S2). Dataset WP, containing all of these loci, had an aligned length of 108 666 bp, among which 14 078 (12.96 %) sites were variable and 7155 (6.58 %) were parsimony-informative (PI). Dataset PCS comprised 72 protein-coding and four rRNA genes; this was 58 063 bp in length, with 6114 variable (10.53 %) and 3067 PI (5.28 %) sites. The proportion of variable and PI sites remained the same or increased slightly when positively selected genes were excluded (datasets WP-ω and PCS-ω). Dataset NCS comprised the 63 non-coding regions with a combined length of 50 603 bp, and the highest proportions of both variable (15.74 %; 7964 total) and PI (8.08 %, 4088 total) sites among datasets (Table 1). Selective pressure analyses showed that four loci (*cemA*, *rpl14*, *rps14* and *rps15*) were estimated to have experienced positive selection. There were ten, 18, five and two positively selected sites (M1a vs. M2a; *P* < 0.05) detected in *cemA*, *rpl14*, *rps14* and *rps15* respectively using the Bayes empirical Bayes (BEB) test. The *cemA* gene has the function of mediating CO_2_ uptake (Wicke *et al.*, 2011). The *rpl14* gene encodes a protein for the small ribosomal subunits, and the *rps14* and *rps15* genes encode large ribosomal subunit proteins, having the function as translation and protein-modifying enzymes (Wicke *et al.*, 2011).

**Table 1.** Comparison of the characteristics in the alignments of different datasets.

Dataset	Length (bp)	Parsimony-informative sites (%)	Variable sites (%)	Identical sites (%)
WP	108 666	7155 (6.58 %)	14 078 (12.96 %)	94 588 (87.04 %)
NCS	50 603	4088 (8.08 %)	7964 (15.74 %)	42 639 (84.26 %)
PCS	58 063	3067 (5.28 %)	6114 (10.53 %)	51 949 (89.47 %)
WP-ω	106 977	7064 (6.60 %)	13 905 (13.00 %)	93 072 (87.00 %)
PCS-ω	56 374	2976 (5.28 %)	5941 (10.54 %)	50 433 (89.46 %)

### Inter- and intra-subgeneric relationships within Rhododendron

**Fig. 1.**
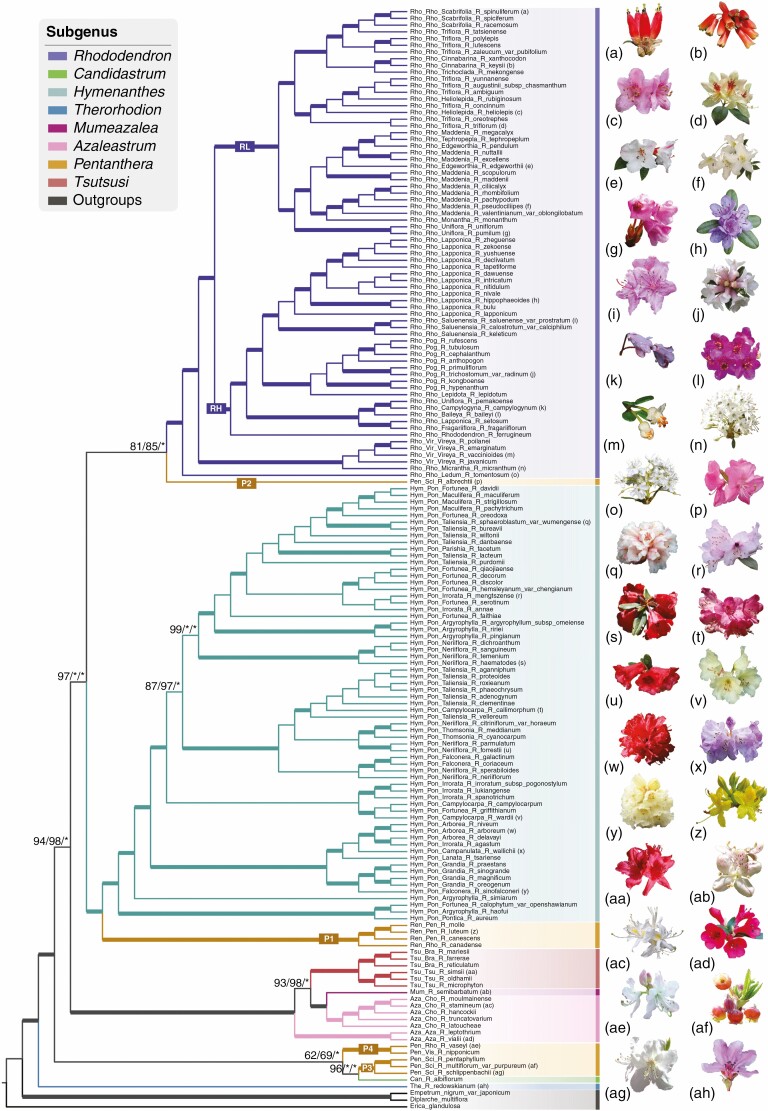
Phylogram of *Rhododendron*. Tree topology is the phylogenetic inference using RAxML for dataset WP. Branches of each subgenus are designated in different colours, and the corresponding subgeneric names are indicated in the key. Tip name contains abbreviations of the subgenus and section, full name of the subsection to which the species belongs, and species name. Support values shown on each branch indicate the phylogeny using RAxML, IQ-TREE and PhyloBayes respectively based on dataset WP. Branches with 100 % BS, 100 % UFBS and 1.0 Bayesian PP values are indicated by thick lines; otherwise, values are indicated along the deep branches (‘*’: 100 % or 1.0). Photographs of *R*. *micranthum* and *R*. *tomentosum* were taken by Mr Ze Wei, *R*. *redowskianum* by Dr Qinwen Lin, *R*. *semibarbatum* by Richard Milne, and the rest by Lianming Gao.

The phylogenetic relationships were highly consistent across all datasets (WP, NCS, PCS-ω and WP-ω) and all tree construction methods (ML and BI) except for PCS (Fig. 1; Supplementary Data Figs S1–11). The phylogenetic relationships based on dataset WP were unaffected by removing positively selected genes (Fig. 1; Figs S1–S3, S8 and S9), but the relationships resolved by the PCS dataset were slightly affected, mainly in the phylogenetic placement of subg. *Candidastrum*, which was sister to subg. *Rhododendron* + *R*. *albrechtii* based on the PCS dataset (Figs S6 and S7) but grouped with parts of subg. *Pentanthera* when positively selected genes were removed, and also in all other datasets (Figs S10 and -S11, and see below).

For the ML trees reconstructed by RAxML, both datasets WP and WP-ω had the highest phylogenetic resolution, with 80 % (128/161) of the internal nodes having bootstrap support (BS) ≥ 90 % (Supplementary Data Figs S1 and S8; Table 2). Dataset NCS had 75 % (121/161) of nodes with BS ≥ 90 % (Fig. S4; Table 2), but datasets PCS and PCS-ω only had 63 % and 62 % nodes with BS ≥ 90 %, respectively (Figs S6 and S10; Table 2). Here only the results from the WP dataset are reported, unless stated otherwise. *Therorhodion* was recovered as the basal group of *Rhododendron* in all analyses. The three largest subgenera – *Rhododendron*, *Hymenanthes* and *Tsutsusi* – were each resolved as monophyletic with strong support (Fig. 1; Figs S1–S9; Table S3), as were the two sections *Tsutsusi* and *Brachycalyx* of subg. *Tsutsusi*.

**Table 2.** The frequency statistics of BS values in the ML tree based on different datasets using RAxML.

Dataset	BS = 100 %	BS ≥ 90 %	BS ≥ 80 %	BS ≥ 75 %	BS ≥ 50 %	BS < 50 %
WP	104 (65 %)	128 (80 %)	138 (86 %)	143 (89 %)	158 (98 %)	3 (2 %)
NCS	86 (53 %)	121 (75 %)	133 (83 %)	139 (86 %)	153 (95 %)	8 (5 %)
PCS	75 (47 %)	101 (63 %)	113 (70 %)	116 (72 %)	145 (90 %)	16 (10 %)
WP-ω	101 (63 %)	128 (80 %)	138 (86 %)	140 (87 %)	157 (98 %)	4 (2 %)
PCS-ω	70(43 %)	100(62 %)	111(69 %)	114 (71 %)	142 (88 %)	19 (12 %)

The values represent the frequency of the BS value falling within each interval.

Subgenera *Azaleastrum* and *Pentanthera* were recovered as polyphyletic, respectively comprising two (its sections *Azaleastrum* and *Choniastrum*) and four (clades P1, P2, P3 and P4, with P2 comprising only *R. albrechtii*, whereas the former genus *Menziesia* fell within clade P3) clades. Section *Choniastrum* was recovered as sister in turn to subg. *Mumeazalea*, subg. *Tsutsusi* and then sect. *Azaleastrum*. The positions and relationships of clades P2, P3 and P4 all varied slightly between certain datasets (Figs 1 and 4; Supplementary Data Figs S1–S11).

Section *Rhododendron* was resolved as polyphyletic due to sections *Pogonanthum* and *Vireya* being embedded within it. Section *Vireya* itself was consistently monophyletic and sister to *R*. *micranthum*, a species of subsect. *Micrantha* in sect. *Rhododendron*. However, some analyses had a monophyletic sect. *Pogonanthum* as sister to *R*. *lepidotum* of sect. *Rhododendron* [i.e. ML trees of datasets WP, NCS and WP-ω (Fig. 1; Supplementary Data Figs S1, S3–S5, S8 and S9)], whereas in others *R*. *lepidotum* was nested within a paraphyletic *Pogonanthum* [BI tree of dataset WP and ML trees of datasets PCS and PCS-ω (Figs S2, S6, S7, S10 and S11)]. Other than these, and sections *Rhodora* and *Sciadorhodion*, all other sections from which more than one species sampled were strongly supported as monophyletic (Fig. 1; Figs S1–S11; Table S3). Notably subsect. *Ledum*, formerly treated as a distinct genus, was strongly supported as sister to the rest of subg. *Rhododendron* in all datasets except PCS-ω [which placed *Ledum* as sister to *R. albrechtii* (Clade P2) and then subg. *Rhododendron*].

### Divergence time estimation

**Fig. 2.**
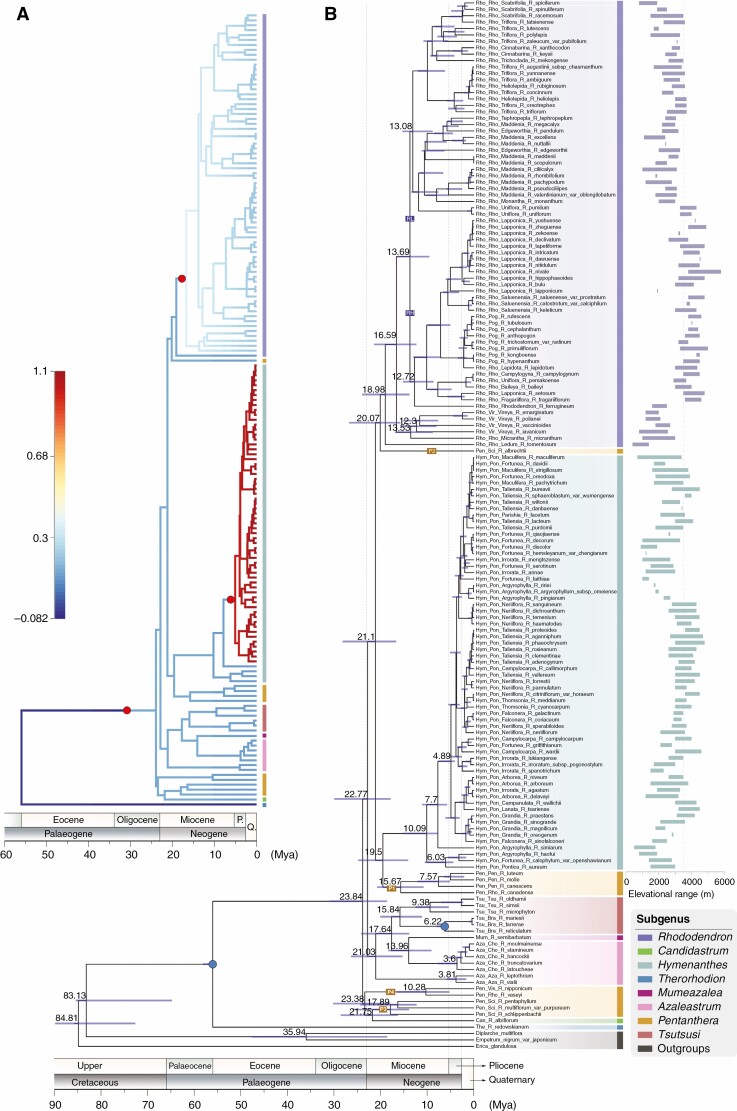
Combined chronogram and phylorate plot of *Rhododendron*. (A) Phylorate plot with branches coloured according to the mean posterior density of net diversification rate (speciation rate minus extinction rate). Blue in the scale represents low rates and red represents high rates. Red circles mark the positions of rate shift in the MAP configuration. (B) Divergence time estimation based on BEAST analysis. The blue bars correspond to the 95 % HPD credibility intervals of age estimates. Nodes with solid blue circles are constrained with fossils.

The divergence time estimates from all of BEAST, RelTime and TreePL analyses were very similar (Fig. 2B; Supplementary Data Figs S12–S14), so only the results from BEAST (Fig. 2B; Fig. S12) are described here. The first divergence, of subg. *Therorhodion*, from the MRCA of all other species occurred 56 Mya [95 % highest posterior density (HPD): 56–58.1 Mya]. After a period of >32 million years (Myr) with no divergence events, a clade comprising *Candidastrum* plus clades P3 and P4 then diverged in the late Oligocene at 23.8 Mya (95 % HPD: 18.6–30.9 Mya). This was the first of a sequence of ten divergence events during the 6.2 Myr between 23.8 and 17.6 Mya, across the Oligocene–Miocene boundary (Fig. 2). Among these, the first nine occurred during a period of 5 Myr, and hence by 18.98 Mya, the following groups had diverged: *Candidastrum*, Clade P3, Clade P4, *Mumeazalea* + subg. *Tsutsusi* + sect. *Choniastrum*, sect. *Azaleastrum*, Clade P1, *Hymenanthes*, Clade P2, subsect. *Ledum*, and all other subg. *Rhododendron* (Fig. 2). There followed a lag of around 10 Myr before crown divergence within *Hymenanthes* (10.1 Mya, 95 % HPD: 7.8–15.2 Mya), following which it diversified very rapidly. Conversely, diversification within subg. *Rhododendron* proceeded at a fairly continuous rate from its origin to the present, with two large but ecologically distinct clades RH (small shrubs occurring mostly >3500 m) and RL (shrubs to small trees occurring mostly < *c*. 3500 m) diverging 13.7 Mya (95 % HPD: 9.5–16.5 Mya) (Fig. 2B). Elsewhere in the tree, subg. *Mumeazalea* diverged from sect. *Choniastrum* at 14 Mya (95 % HPD: 9.1–18.4 Mya), after their MRCA diverged from subg. *Tsutsusi* 17.6 Mya (95 % HPD: 13.9–24.2 Mya). Species of sect. *Choniastrum* began to diversify in the Pliocene at 3.6 Mya (95 % HPD: 3.4–7.7 Mya). Within subg. *Tsutsusi*, the split between sections *Brachycalyx* and *Tsutsusi* was dated to 15.8 Mya (95 % HPD: 11.1–20.1 Mya) in the middle Miocene. Additionally, diversification within sections *Pogonanthum*, *Vireya* and *Pentanthera* initiated at 2.1 Mya (95 % HPD: 1.5–3.3 Mya), 12.3 Mya (95 % HPD: 8–15.7 Mya) and 7.6 Mya (95 % HPD: 5.1–12.1 Mya), respectively. The divergence between the two remaining lineages, *R*. *nipponicum* and *R*. *vaseyi*, occurred at 10.3 Mya (95 % HPD: 5.2–16.6 Mya).

### Diversification analyses

**Fig. 3.**
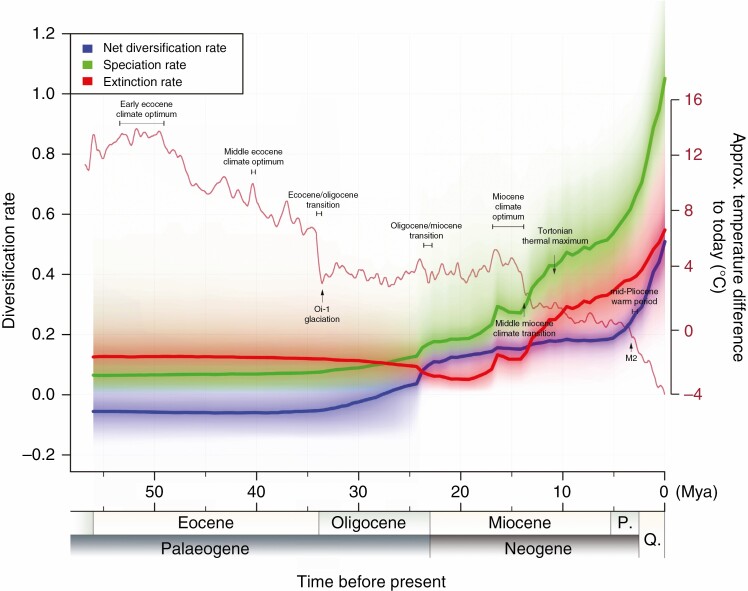
Rate-through-time plots for speciation, extinction and net diversification with 95 % confidence intervals indicated by shaded areas. Approximate annual air temperature differences from the present day are derived from Westerhold *et al.* (2020).

The phylorate plot from BAMM analysis indicated that the net diversification rate varied from low to high within *Rhododendron* (Fig. 2A). In total, three significant rate accelerations were detected (Fig. 2A). One was crown diversification of all *Rhododendron* except subg. *Therorhodion*, the second within subg. *Rhododendron* soon after its origin (*c*. 16.6 Mya) and the third within *Hymenanthes* but much later – *c*. 4.9 Mya, and hence around 14 Myr after its origin. The rate-through-time plot suggested that the net diversification, speciation and extinction rates were fairly constant up to 36 Mya, at which point the diversification and speciation rates began climbing slowly, then had a brief but substantial increase at ~24 Mya, after which the diversification rate climbed slowly until a remarkable acceleration from 5 Mya to the present. Meanwhile the speciation rate climbed slowly, followed by a significant increase and then a slight decline between 17 and 14 Mya, and then climbed rapidly until a remarkable acceleration from 5 Mya to the present. The extinction rate declined slowly from 36 to 20 Mya, and was indicated to be higher than the speciation rates until around 28 Mya, then from 20 to 5 Mya it tracked the speciation rate upwards, while always remaining >0.1 below it (Fig. 3). For the last 5 Myr it ceased to keep pace with speciation, leading to a steady increase in the net diversification rate from then to the present. The diversification rate shifts detected were concordant between the rate-through-time and the phylorate plot (Figs 2A and 3).

The LTT plot generated similar results as BAMM analysis and showed an accumulation of lineages since the late Oligocene, *c*. 24 Mya (Supplementary Data Fig. S15). In MEDUSA analysis, the background net diversification rate for *Rhododendron* was estimated as 0.0301 spp./Myr, and four significant changes of diversification rate were detected, comprising three increases and one decrease (Fig. S16). An increase from 0.0301 to 0.2343 spp./Myr occurred at crown divergence of the clade comprising all *Rhododendron* species except *Therorhodion*, *Candidastrum*, Clade P3 and Clade P4, then within this clade a further increase to 0.3558 spp./Myr was detected in the clade comprising subg. *Rhododendron* excluding subsect. *Ledum*. Elsewhere, an increase from 0.0301 to 0.1303 spp./Myr was detected within Clade P3. The detected decrease in the diversification rate, in Clade P2, involved drops in the rate from 0.2343 to 0 spp./Myr (Fig. S16).

For subg. *Rhododendron*, the mean net diversification rate was 0.1817 spp./Myr and was barely affected by the inclusion or exclusion of *Vireya* and/or *Ledum* (Supplementary Data Table S4); however, its mean species age of 2.81 drops to 2.29 Myr when both are excluded, with intermediate values when either one is excluded. Likewise, the mean net diversification rate and mean species age for *Hymenanthes* were 1.0156 spp./Myr and 0.98 Myr, whereas when the Tertiary relict species of subsect. *Pontica* were excluded the former increased marginally to 1.0292 spp./Myr whereas the latter dropped to 0.91 Myr. Hence when non Hengduan–Himalayan groups were excluded, then relative to *Rhododendron* the net diversification rate of *Hymenanthes* was more than five times faster, and its species age on average more than 60 % younger. The mean species age of clades within subg. *Rhododendron* showed that Clade RH (small shrubs, high elevation; 1.38 Myr) was younger than Clade RL (shrubs or small trees, relatively low elevation; 2.77 Myr), but the mean diversification rates of clades RH and RL were similar (0.1896 vs. 0.1780 spp./Myr).

## DISCUSSION

### Intensive sampling produces high resolution but reveals phylogenetic conflicts

Based on extensive sampling across taxa and the chloroplast DNA (cpDNA) genome, the full plastid dataset produced a well-resolved and well-supported phylogeny, yet several nodes were conflicted by partial datasets, and many conflicted with previous studies based on the *matK* region (Kurashige *et al.*, 2001; Khan *et al.*, 2021). However, very few topological conflicts existed between the different phylogenetic analysis methods used on our datasets, and most of the conflicting topologies were weakly supported.

**Fig. 4.**
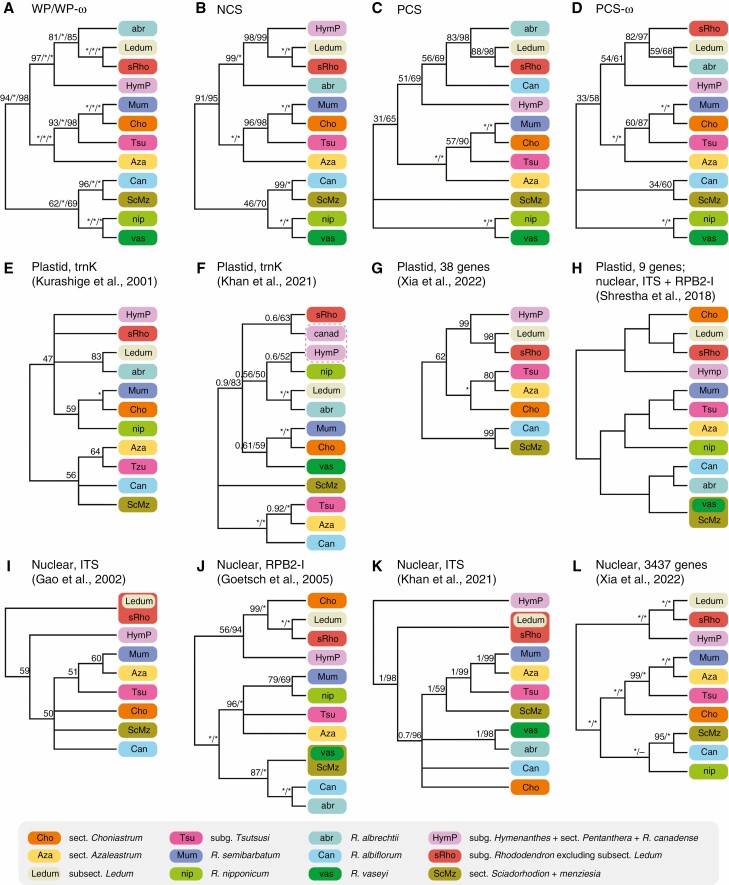
Comparisons of phylogenetic relationships of core *Rhododendron* between our analyses (A–D) and previous studies (E–L). In cases where multiple support values are shown, these are from different analysis methods and stated in the order they are mentioned for each tree, with values of 100 or 1 represented by an asterisk (*). (A) Phylogenetic relationships inferred from dataset WP using RAxML, PhyloBayes and IQ-TREE, which are also recovered from dataset WP-ω using RAxML and IQ-TREE; (B–D) phylogenetic relationships inferred from datasets NCS, PCS and PCS-ω respectively using RAxML and IQ-TREE; (E) phylogenetic relationships based on *matK* and *trnK* intron using PAUP (MP tree) summarized from figure 3 in Kurashige *et al.* (2001); (F) phylogenetic relationships based on *trnK* using MrBayes and IQ-TREE summarized from figure 1 in Khan *et al.* (2021); (G) phylogenetic relationships based on 38 plastid genes using IQ-TREE summarized from figure S3 in Xia *et al.* (2022); (H) phylogenetic relationships based on nine chloroplast genes plus ITS and RPB2-I regions using BEAST summarized from supporting information appendix S5 in Shrestha *et al.* (2018); (I) phylogenetic relationships based on ITS using PAUP (MP tree) summarized from figure 1 in Gao *et al.* (2002); (J) phylogenetic relationships based on RPB2-I using PAUP (MP tree) and MrBayes summarized from figure 2 in Goetsch *et al.* (2005); (K) phylogenetic relationships based on ITS using MrBayes and IQ-TREE summarized from figure 2 in Khan *et al.* (2021); (L) phylogenetic relationships based on 3437 nuclear orthologous genes using IQ-TREE and ASTRAL summarized from figures S1 and S2 in Xia *et al.* (2022).

Comparing the current analysis with all past analyses (Fig. 4), relatively few relationships are constant across all analyses, but subg. *Therorhodion* is always undisputedly sister to all other *Rhododendron*, and here the genus excluding *Therorhodion* is termed ‘core *Rhododendron*’ for ease of discussion. Subgenus *Pentanthera* (*sensu*Chamberlain *et al.*, 1996) was highly polyphyletic, whereas sect. *Pentanthera* plus *R*. *canadense* is always monophyletic and sister to subg. *Hymenanthes*. The two sections of subg. *Azaleastrum* (*Azaleastrum* and *Choniastrum*) are each always monophyletic but never sister to one another. Species from sect. *Sciadorhodion* of subg. *Pentanthera* (other than *R. albrechtii*) formed a clade here termed the *ScMz* clade, also including the former genus *Menziesia* (see also Goetsch *et al.*, 2005; Craven, 2011; Xia *et al.*, 2022). Subgenus *Tsutsusi* is always monophyletic as well as its two sections *Tsutsusi* and *Brachycalyx*. Subgenus *Rhododendron* is always monophyletic except that cpDNA sometimes places the former genus *Ledum* outside it (Supplementary Data Figs S10 and S11; Kurashige *et al.*, 2001; Khan *et al.*, 2021). The relationships of five individual species are inconsistent across all studies: these are *R. vaseyi*, *R. nipponicum*, *R. albrechtii* (all belonging to subg. *Pentanthera sensu*Chamberlain *et al.*, 1996), *R. albiflorum* (the monotypic subgenus *Candidastrum*) and *R. semibarbatum* (the monotypic subgenus *Mumeazalea*). Therefore, higher level relationships in core *Rhododendron* can be described across studies in terms of 12 clades of greatly varying sizes (Fig. 4): *R. vaseyi*, *R. nipponicum*, *R. albrechtii*, *R. albiflorum*, *R. semibarbatum*, the *ScMz* clade, *Hymenanthes* + sect. *Pentanthera*, subg. *Rhododendron* excluding *Ledum*, former genus *Ledum* (merged into *Rhododendron* by Kron and Judd, 1990), subg. *Tsutsusi*, sect. *Azaleastrum* and sect. *Choniastrum*. For ease of discussion, the last six are henceforth referred to as *HymP*, *sRho*, *Ledum*, *Tsutsusi*, *Azaleastrum* and *Choniastrum*. Many of these clades have already been recognized or suggested at the subgenus level (Chamberlain *et al.*, 1996; Gao *et al.*, 2002; Goetsch *et al.*, 2005; Fu *et al.*, 2022), but here we tentatively suggest that all 12 might ultimately merit recognition at this rank, once adequate data become available.

Relationships among these 12 core *Rhododendron* groups were fully resolved and generally well supported in our full (WP) plastid dataset. However, the position of *R. albrechtii* was altered relative to WP in the NCS and PCS-ω (but not PCS) datasets, and that of *R. albiflorum* shifted in the PCS (but not PCS-ω) dataset. Hence the positions of both species are sensitive to the inclusion or exclusion of genes under selection that might be subject to homoplasious adaptative changes (Fig. 4A–D; Supplementary Data Figs S6, S7, S10 and S11), and the differences involving the PCS and PCS-ω datasets are generally not strongly supported. However, regarding the conflict between NCS and WP, support for *R. albrechtii* branching before *HymP* is near maximum under NCS, but the reverse relationship has 81–85 % BS/UFBS support in the WP dataset, and slightly more with genes under selection removed (WP-ω; 82–87 % BS/UFBS). Therefore, coding genes not under detectable selection are responsible for the difference, and it is unclear which relationship better reflects the true plastid tree.

Our study strongly supported a clade of (*Azaleastrum* (*Tsutsusi* (*Choniastrum* + *R*. *semibarbatum*))), and generally this clade was consistent (Fu *et al.*, 2022) or showed few conflicts (Xia *et al.*, 2022) with recent phylogenies that sampled widely across the plastome and densely across taxa. Conversely, there were strong conflicts with previous phylogenies based on the plastid *matK* region (Kurashige *et al.*, 2001; Khan *et al.*, 2021), or on multiple cpDNA regions plus nuclear genes (Shrestha *et al.*, 2018), mainly concerning the placement of *Tsutsusi* + *Azaleastrum* as sister to *R*. *albiflorum*, whereas *Choniastrum* + *R*. *semibarbatum* was sister to *R*. *vaseyi* or *R*. *nipponicum* but with weak support, hence breaking up groupings that are strongly supported in the current study. These *matK*-based analyses concurred with our PSC-ω dataset in placing *R*. *albrechtii* sister to *Ledum* (Fig. 4D).

These findings indicate strongly that a phylogeny based on a single plastid region, or even many, cannot be assumed to represent the true plastid tree, and even casts doubt on whether such a thing exists. The most well-supported discordance in our own datasets, concerning the position of *R. albrechtii* between our WP and NCS datasets, might result from plastid recombination, albeit probably involving more than one or two genes. An alternative hypothesis of incomplete lineage sorting cannot explain how this species appears in completely different clades in nuclear phylogenies, whereas both phylogenies are consistent with a past hybridization event.

Of nuclear phylogenetic studies of the whole genus, Xia *et al.* (2022) sampled by far the most of the genome, i.e. 3437 nuclear orthologous genes from transcriptome data, whereas others used single regions, i.e. RPB2 (Goetsch *et al.*, 2005) or ITS (Gao *et al.*, 2002; Khan *et al.*, 2021). The positions of *Choniastrum* and (where included) each of *R*. *albrechtii*, *R*. *vaseyi* and *R*. *nipponicum* vary dramatically between these studies. If these four lineages are all removed, then our study (except dataset PCS), Xia *et al.*’s (2022) plastids and all these nuclear only analyses would resolve the same three clades: (*HymP* (s*Rho* + *Ledum*)), (*Azaleastrum* + *Mumeazalea* + *Tsutsusi*) and (*Candidastrum* + *ScMz*). However, the former two are sister for all our plastome datasets, whereas the latter two are sister in all four nuclear studies, strongly indicating a reticulation event in the genus’ deep history. Together with all the other instances of discordance noted here, it seems very likely that numerous reticulate evolutionary events occurred during the history of this genus, and there can be no single correct species tree for it. Many of the five single species that have variable positions between phylogenies (*R. albrechtii*, *R. albiflorum*, *R*. *semibarbatum*, *R. vaseyi* and *R. nipponicum*) might have hybrid origins, and it is important that all of these are included in all future genus-level phylogenetic analyses if these issues are to be resolved.

The species barrier within *Rhododendron* is very fragile and numerous natural hybridization events have been detected (Milne *et al.*, 1999, 2010; Zhang *et al.*, 2007; Zha *et al.*, 2008, 2010; Ma *et al.*, 2010; Yan *et al.*, 2017, 2019; Zheng *et al.*, 2021). Hybridization/introgression will result in shared maternally inherited genotypes between closely related species (Du *et al.*, 2009), which may lead to conflicts between nuclear and plastid phylogenies. Xia *et al.* (2022) obtained a well-resolved phylogeny based on 3437 orthologous nuclear genes, but some species relationships still conflicted with those inferred from plastid sequences in their study and the present study. However, they had issues with missing data in the 38 plastid protein-coding genes, and some key species were missing from their plastid analysis. Our phylogeny represented all subgenera and sections but only 35 of 59 *Rhododendron* subsections (*c*. 59 %), and ~45 % of *Rhododendron* species present in the Himalaya–Hengduan Mountains were sampled. Hence denser sampling of taxa, examining both organelle and nuclear genomes, is needed to better understand the divergence and diversification history of *Rhododendron*.

### Divergence time and diversification history

We obtained a younger estimated age of diversification for most extant lineages as compared with Xia *et al.* (2022) and Shrestha *et al.* (2018). All three methods used (BEAST, Reltime and treePL) gave very similar results (Fig. 2B; Supplementary Data Figs S12–S14), indicating that sensitivity to the method used becomes small when enough taxa and genomes are sampled. Hence the results discussed here are from BEAST unless stated otherwise. However, compared to Xia *et al.* (2022), who also used Reltime with high taxon and genome coverage, we had fewer taxa but many more plastid protein-coding genes and in particular included non-coding regions.

We estimated the crown age of *Rhododendron* (i.e. divergence of *Therorhodion*) at 56 Mya, as inferred by Rose *et al.* (2018) and Xia *et al.* (2022). Fossil evidence indicates that early lineages of *Rhododendron* went extinct before this, during the Cretaceous–Palaeogene mass extinction event (Collinson and Crane, 1978), and the above date indicates that all extant taxa derive from a single surviving lineage. Crown divergence of core *Rhododendron* from our data was >30 Myr later, around the Oligocene–Miocene boundary at 23.8 Mya (Fig. 2B), a little older than Rose *et al.*’s (2018) 18.3 Mya estimation, but much younger than the 35.9 Mya estimation of Xia *et al.* (2022); the ~56 Mya estimation of Shrestha *et al.* (2018) appears to be an outlier.

Our data indicate that, during a brief 6.2-Myr period from 23.8 to 17.6 Mya (Fig. 2B), coinciding with climate cooling and intensification of the Asian summer monsoon around the Oligocene–Miocene transition (Deng *et al.*, 2019; Su *et al.*, 2019; S. F. Li *et al.*, 2021), core *Rhododendron* diversified from one into ten lineages. Eight of the 12 component clades listed above had split, and *HymP* had itself split into deciduous and evergreen clades. Of the other four, *R. semibarbatum* diverged from *Choniastrum* at 13.96 Mya and *R. nipponicum* from *R. vaseyi* at 10.28 Mya. Of course, this is not the complete picture as hybridization events not detectable from this data were probably involved too. For example, here *Mumeazalea* diverged from *Choniastrum* 1.88 Myr after crown divergence in *Tsutsusi*, whereas Xia *et al.*’s (2022) nuclear data have it diverging from *Azaleastrum* earlier than crown divergence in *Tsutsusi* – hence a hypothesis to test is that it derived from a cross between sister lineages of *Choniastrum* and *Azaleastrum.*

Unsurprisingly given this rapid expansion of lineage numbers, crown divergence in core *Rhododendron* formed the first of three significant increased rate shifts in *Rhododendron* diversification detected by BAMM analysis (Fig. 2A), with the rate-through-time plot giving similar results (Fig. 3). The other two shifts were detected in the species-rich subgenera *Hymenanthes* and *Rhododendron*. The rate shift in subg. *Rhododendron* occurred *c*. 16.6 Mya when the species of the *sRho* clade began to diversify, after which Clade RH diverged from Clade RL at 13.7 Mya. This might have been an ecological speciation event, because Clade RH comprises small, narrow-leaved shrubs of thickets or open alpine habitats mostly above 3500 m, whereas Clade RL comprises larger leaved shrubs/small trees from in or around forests below 3500 m. This coincides with the Himalayas nearing present-day elevations at *c*. 17–14 Mya, driven by ongoing tectonic events (Wang *et al.*, 2012; Su *et al.*, 2019; Ding *et al.*, 2020), generating complex terrain and heterogeneous habitats. Subsequent diversification in both clades might have been promoted by ongoing orogeny (Kapp and DeCelles, 2019), the intensification of the Asian summer monsoon in the Himalaya–Hengduan Mountains from ~14 Mya onwards (Farnsworth *et al.*, 2019; S. F. Li *et al.*, 2021; Spicer *et al.*, 2021), and increasing moisture availability, leading to deeper valleys through river incision (Wang *et al.*, 2012; Nie *et al.*, 2018). All this would have promoted habitat diversity and barriers to dispersal, promoting parallel speciation in both clades.

Despite their similar mean net diversification rate (0.1896 vs. 0.1780 spp./Myr), the average species age in Clade RH is younger than in RL (1.38 vs. 2.77 Myr), indicating more recent radiation within Clade RH, which could be because their alpine habitats were only recently generated by mountain uplift and Quaternary global cooling (Ding *et al.*, 2020). However, the mean divergence age across the whole of *Hymenanthes* was even younger (0.98 Myr), and it has a higher mean net diversification rate (1.0292 vs. 0.1827 spp./Mya) than subg. *Rhododendron* in the Himalaya–Hengduan Mountains. Hence despite both subgenera having a clear centre of diversity in this region, the timing and manner of diversification clearly differs between them. Both *Hymenanthes* and *sRho* diverged from their sister groups around 19.5 Mya, but while diversification in *sRho* was fairly continuous, crown divergence in *Hymenanthes* did not initiate until ~10 Mya (Fig. 2B; Milne, 2004). Furthermore, the first diverging clade of *Hymenanthes* comprises low-altitude Tertiary relict species (mostly not sampled here but see Milne, 2004; Milne *et al.*, 2010) with a nested NE Himalayan subclade. Therefore, *Hymenanthes* may not have entered the Himalaya until after this clade diverged, hence much later than subg. *Rhododendron*. Furthermore, the next diverging species (*R*. *simiarum* at *c*. 7.7 Mya) is also a low-altitude species. The rate of diversification increased significantly *c*. 4.9 Mya according to BAMM analysis, with most species diverging after that (Fig. 2B; Milne, 2004). This sudden acceleration of diversification might have resulted from its invasion of the Himalaya region. Other possible contributors around that time include gradual global cooling (Milne, 2004; Milne and Abbott, 2002), and a period of high monsoon intensification (Ding *et al.*, 2020; Xia *et al.*, 2022), which together facilitated ecological and evolutionary opportunities for diversification in other groups (Luo *et al.*, 2016; Ye *et al.*, 2019). Hence, although a few clades in *Hymenanthes* are high-altitude only, overall altitudinal preference appears more plastic in *Hymenanthes* than in subg. *Rhododendron* despite the former having diversified over a shorter period.

Compared to our results, the best nuclear data available (Xia *et al.* 2022) indicate that crown diversification in core *Rhododendron* began considerably earlier, around 36 Mya, and diversification within the *Tsutsusi*–*Azaleastrum*–*Choniastrum*–*Mumeazalea*–*ScMz*–*R*. *nipponicum*–*R*. *vaseyi*–*Candidastrum* clade has proceeded at a steady rate since then. Early nodes involving subgenera *Hymenanthes* and *Rhododendron* are likewise around 8.8–10.3 Myr older than ours. Consequently, their analysis allows more time for diversification, and so rate shifts are much less apparent.

## CONCLUSIONS AND FUTURE DIRECTIONS

*Rhododendron* is a large genus that is taxonomically difficult for two reasons. The first issue, recent rapid radiation, means that some clades may be supported by only a few apomorphic markers, and hence wide genomic coverage, as in this paper and Xia *et al.* (2022), will be necessary to resolve some clades, especially within *Hymenanthes* where much of the radiation has been very recent (Fig. 2B; Milne, 2004). Second, hybridization is rampant, and discordance between phylogenies based on different markers indicates that multiple reticulate evolution events may have occurred, and that no single marker can reconstruct the true species tree. Our phylogeny, sampling heavily across both taxa and the plastid genome, provides a major advance, yet also indicates that recombination might have occurred, due to hybridization/introgression, even within the plastid genome.

The identification of clades at both higher and lower levels that are consistently monophyletic across all markers and analyses is an important step towards unravelling *Rhododendron* evolution. The 12 clades of core *Rhododendron* identified here represent a step towards this, but even some of these are challenged by certain analyses, though this could occur due to undersampling of the genome (e.g. *Ledum* nests within *sRho* for ITS; Gao *et al.*, 2002; Khan *et al.*, 2021), or very uneven marker sampling across taxa (as in Shrestha *et al.*, 2018). A study that samples all 12 clades with at least the nuclear genome coverage of Xia *et al.* (2022) is urgently needed, and from such data it would be possible to test which clades are retained when different portions of the nuclear genome are sampled. With clades demonstrated, or even tentatively assumed, to be monophyletic, then approaches such as integrated single copy gene (SCG) trees and phylonet-based network analysis (e.g. M. J. Li *et al.*, 2021) can be used to begin to uncover patterns of reticulate evolution, and hence identify clades of hybrid origin.

Numerous natural hybridization events have been detected, and hence populations sampled for phylogenetic analysis (either directly or via material taken for cultivation) might have acquired cpDNA or nuclear material from other species. Therefore, sampling of multiple populations from different points in each species’ range is desirable where possible (Wang *et al.*, 2022). While this will increase the resources required for sampling, species can be pruned to one individual for phylogenetic analysis if no introgression is detected.

Comparing the current study with Xia *et al.* (2022), clade ages throughout the genus seem to differ depending on which genome is examined, in spite of wide sampling of both taxa and genomes. More research is needed to determine why this difference exists, before truly reliable node age estimates can be obtained. Nonetheless, both studies found that *Hymenanthes* began to diversify 7–9 Myr after subg. *Rhododendron*, but diversified faster, so despite the two subgenera both having centres of diversity in and around the eastern Himalaya, it is clear that they did not diversify simultaneously. Our data indicate that highly heterogeneous habitats caused by active orogeny, plus climate cooling and the intensification of the Asian summer monsoon from the late Oligocene onwards were probably significant for diversification in subg. *Rhododendron*, whereas *Hymenanthes* might have invaded the mountains late in their history and radiated as a result. The two subgenera were also shown to differ in the ecological patterns of their divergence, with far more transitions between high and low altitudes in *Hymenanthes* than in *Rhododendron*. Studies like these will help with the development of a stable and reliable taxonomic framework for *Rhododendron*, as well as help us to understand what drove its diversification and ecological adaption, all of which will aid the conservation of *Rhododendron*.

## SUPPLEMENTARY DATA

Supplementary data are available online at https://academic.oup.com/aob and consist of the following. Table S1. Taxa included in this study with classification, locality and voucher information. Table S2. Genes and intergenic regions recovered in the sampled taxa. Table S3. Summary of the monophyly and corresponding support values of subgenera, sections and subsections in *Rhododendron* with multiple sampled species by phylogenetic analyses. Table S4. Mean net diversification rate and species age of the clades in subgenera *Rhododendron* and *Hymenanthes*. Figure S1. ML tree inferred from dataset WP using RAxML. Figure S2. BI tree inferred from dataset WP using PhyloBayes. Figure S3. ML tree inferred from dataset WP using IQ-TREE. Figure S4. ML tree inferred from dataset NCS using RAxML. Figure S5. ML tree inferred from dataset NCS using IQ-TREE. Figure S6. ML tree inferred from dataset PCS using RAxML. Figure S7. ML tree inferred from dataset PCS using IQ-TREE. Figure S8. ML tree inferred from dataset WP-ω using RAxML. Figure S9. ML tree inferred from dataset WP-ω using IQ-TREE. Figure S10. ML tree inferred from dataset PCS-ω using RAxML Figure S11. ML tree inferred from dataset PCS-ω using IQ-TREE. For figures S1 to S11, support values are given on branches. Figure S12. Divergence times of *Rhododendron* estimated from dataset WP using BEAST. Figure S13. Divergence times of *Rhododendron* estimated from dataset WP using treePL. Figure S14. Divergence times of *Rhododendron* estimated from dataset WP using RelTime. Figure S15. LTT plots in *Rhododendron*. Figure S16. Diversification patterns of major lineages inferred from MEDUSA analyses based on the MCC tree from BEAST analysis.

mcac114_suppl_Supplementary_Figure_S1

mcac114_suppl_Supplementary_Figure_S2

mcac114_suppl_Supplementary_Figure_S3

mcac114_suppl_Supplementary_Figure_S4

mcac114_suppl_Supplementary_Figure_S5

mcac114_suppl_Supplementary_Figure_S6

mcac114_suppl_Supplementary_Figure_S7

mcac114_suppl_Supplementary_Figure_S8

mcac114_suppl_Supplementary_Figure_S9

mcac114_suppl_Supplementary_Figure_S10

mcac114_suppl_Supplementary_Figure_S11

mcac114_suppl_Supplementary_Figure_S12

mcac114_suppl_Supplementary_Figure_S13

mcac114_suppl_Supplementary_Figure_S14

mcac114_suppl_Supplementary_Figure_S15

mcac114_suppl_Supplementary_Figure_S16

mcac114_suppl_Supplementary_Table_S1

mcac114_suppl_Supplementary_Table_S2

mcac114_suppl_Supplementary_Table_S3

mcac114_suppl_Supplementary_Table_S4
