# Supplementary figures and images for: Resolution, conflict and rate shifts: insights from a densely sampled plastome phylogeny for Rhododendron (Ericaceae)
Source: Ann Bot. 2022 Sep 10;130(5):687–701. doi: 10.1093/aob/mcac114 (PMC9670778; doi:10.1093/aob/mcac114)

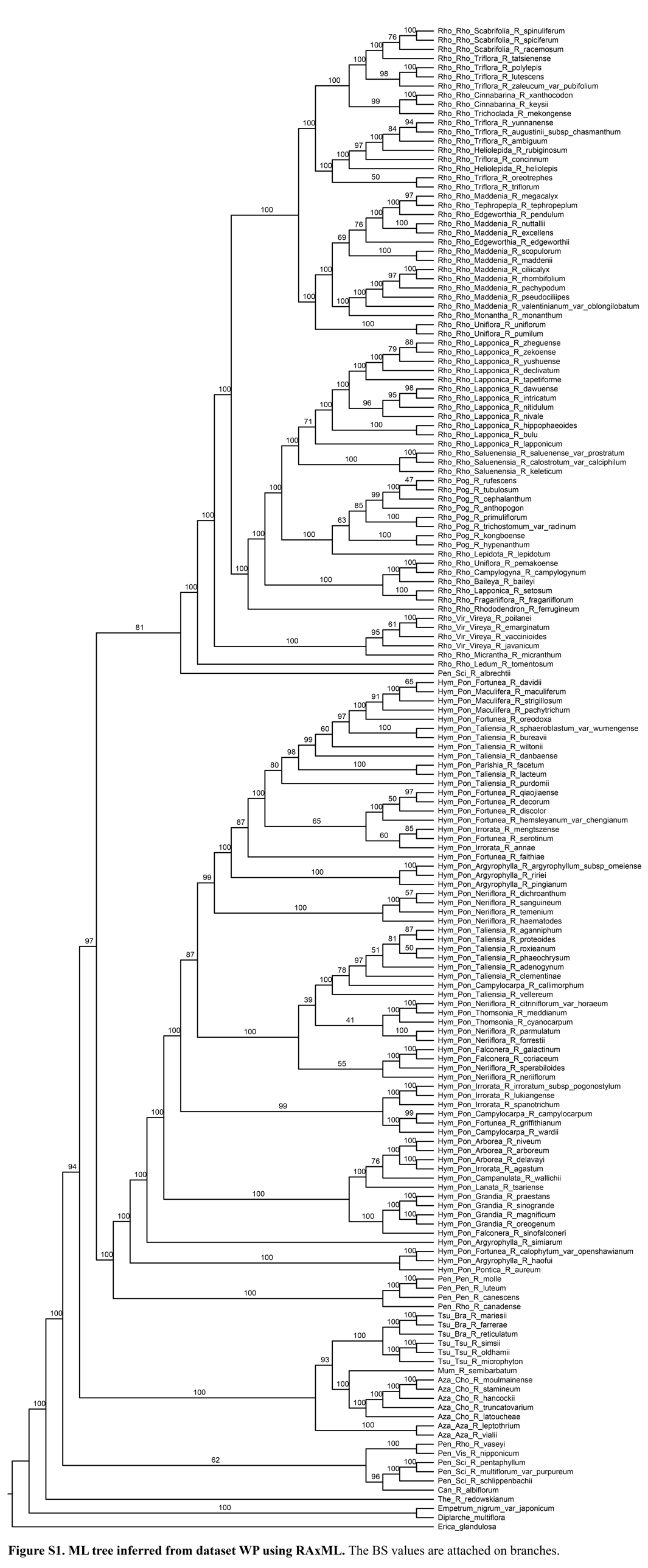

Supplement: mcac114_suppl_Supplementary_Figure_S1 [file mcac114_suppl_supplementary_figure_s1.jpeg]

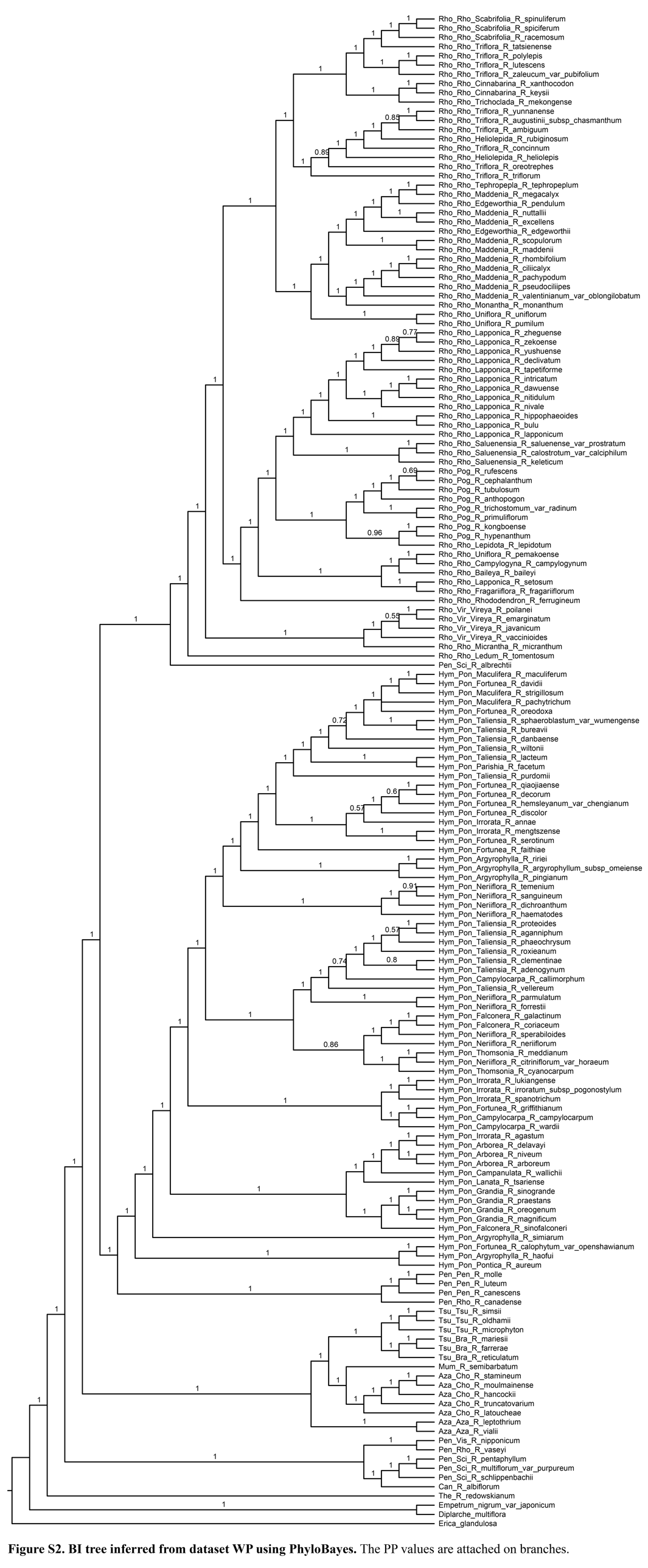

Supplement: mcac114_suppl_Supplementary_Figure_S2 [file mcac114_suppl_supplementary_figure_s2.jpeg]

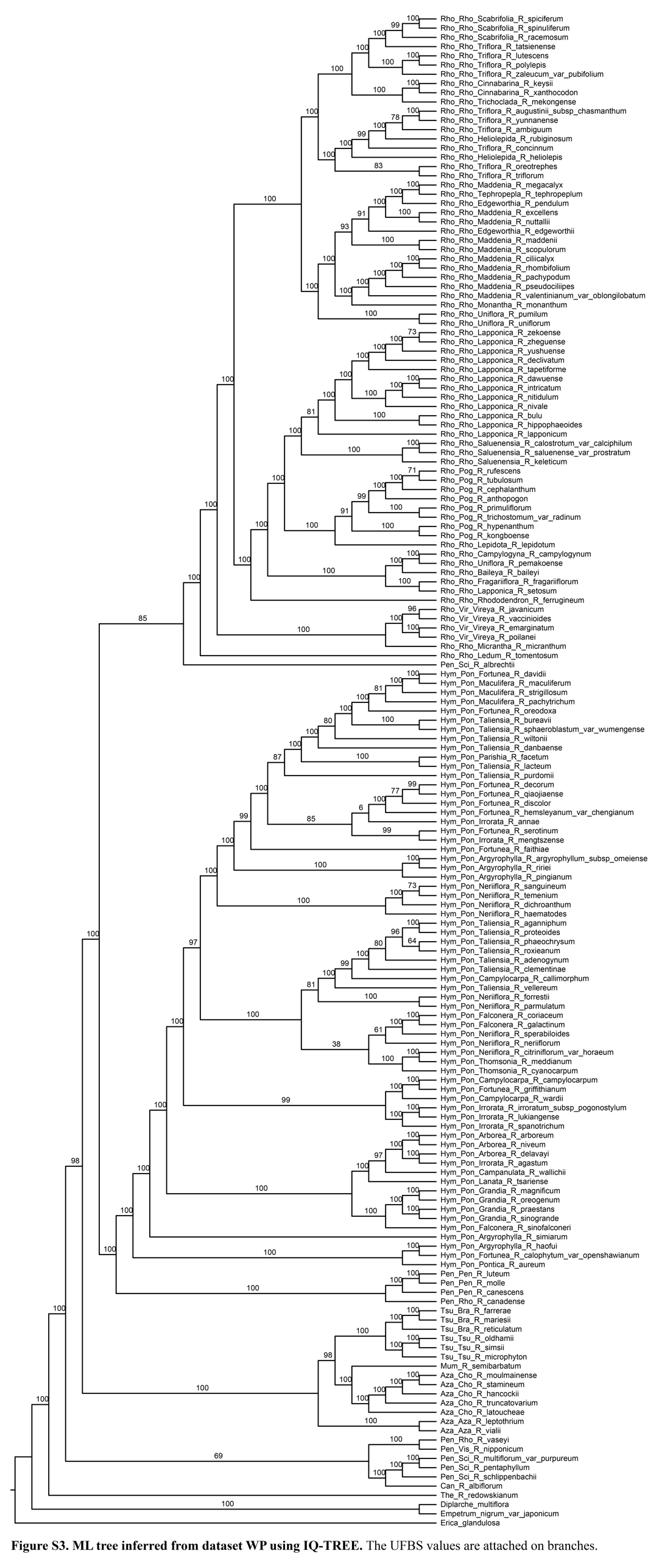

Supplement: mcac114_suppl_Supplementary_Figure_S3 [file mcac114_suppl_supplementary_figure_s3.jpeg]

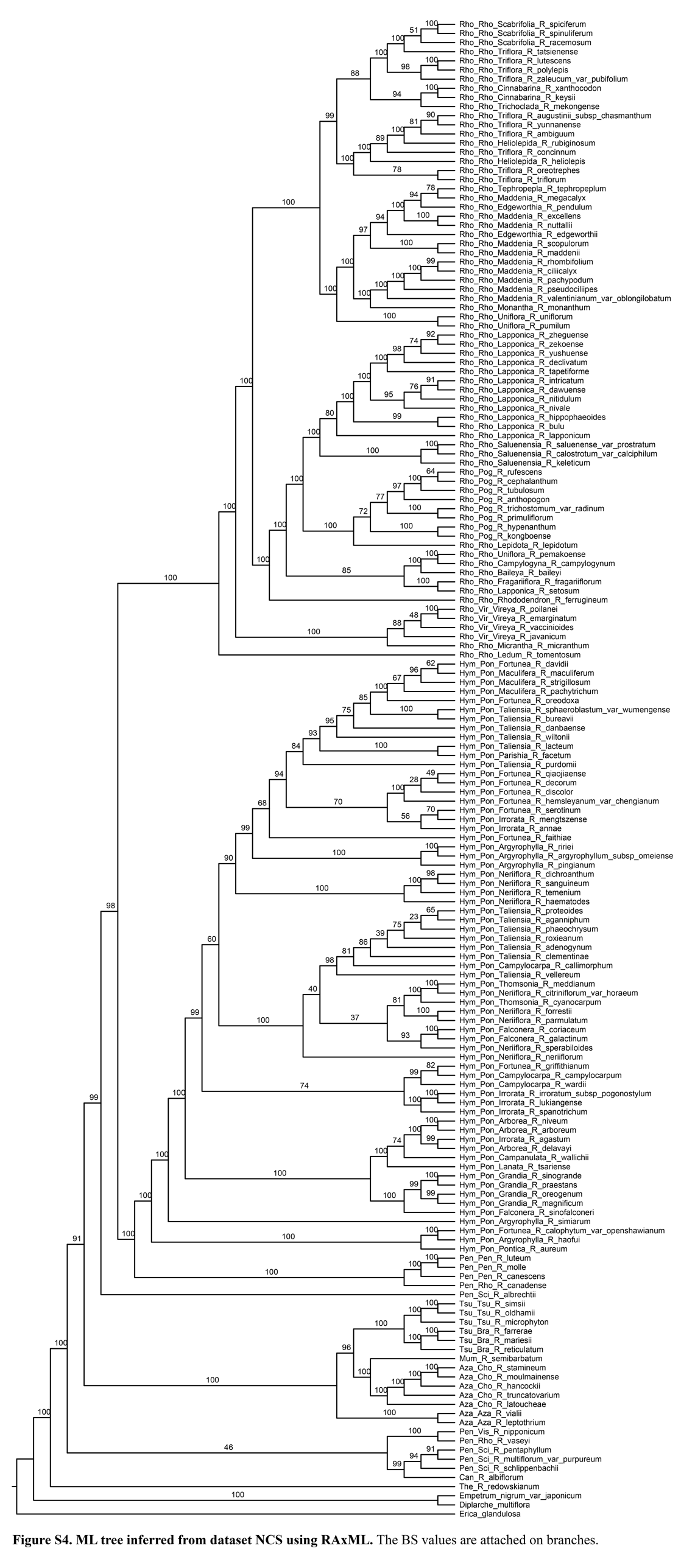

Supplement: mcac114_suppl_Supplementary_Figure_S4 [file mcac114_suppl_supplementary_figure_s4.jpeg]

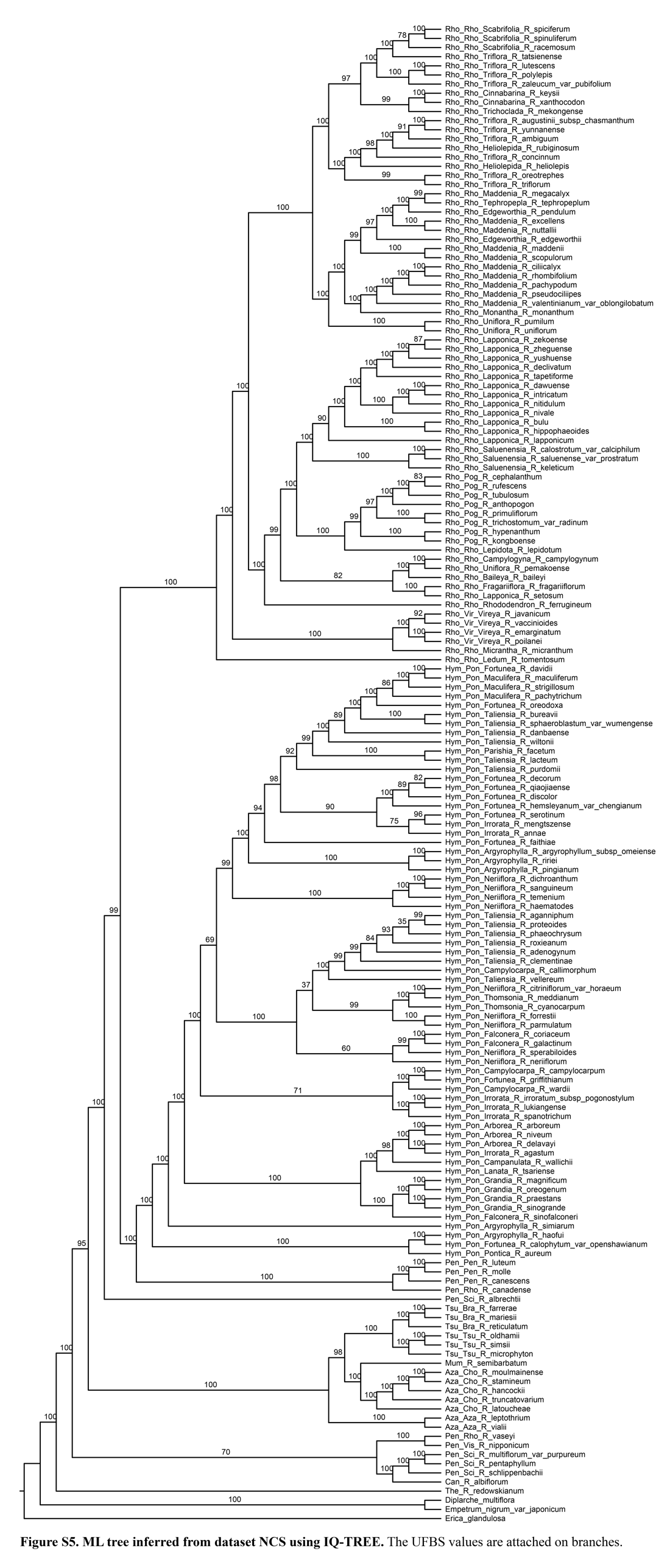

Supplement: mcac114_suppl_Supplementary_Figure_S5 [file mcac114_suppl_supplementary_figure_s5.jpeg]

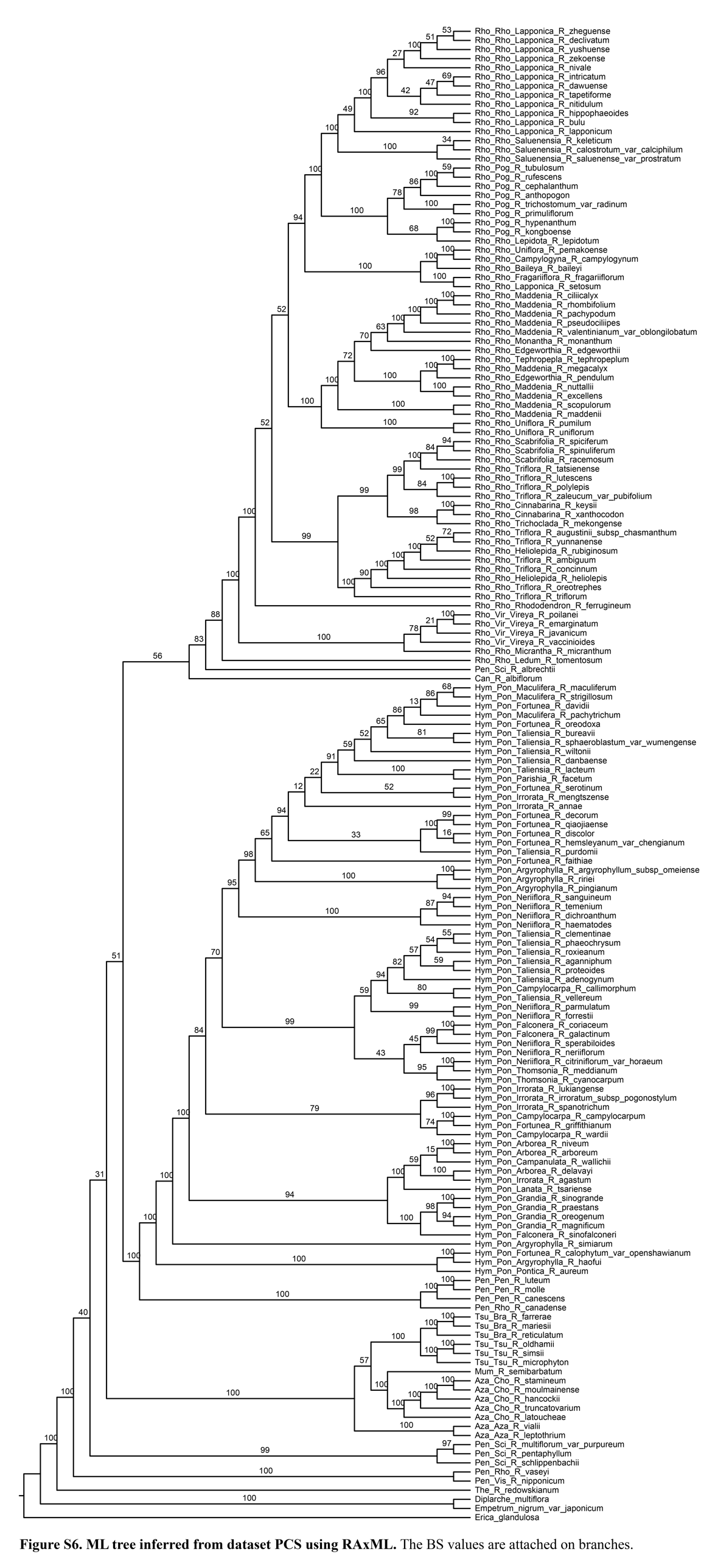

Supplement: mcac114_suppl_Supplementary_Figure_S6 [file mcac114_suppl_supplementary_figure_s6.jpeg]

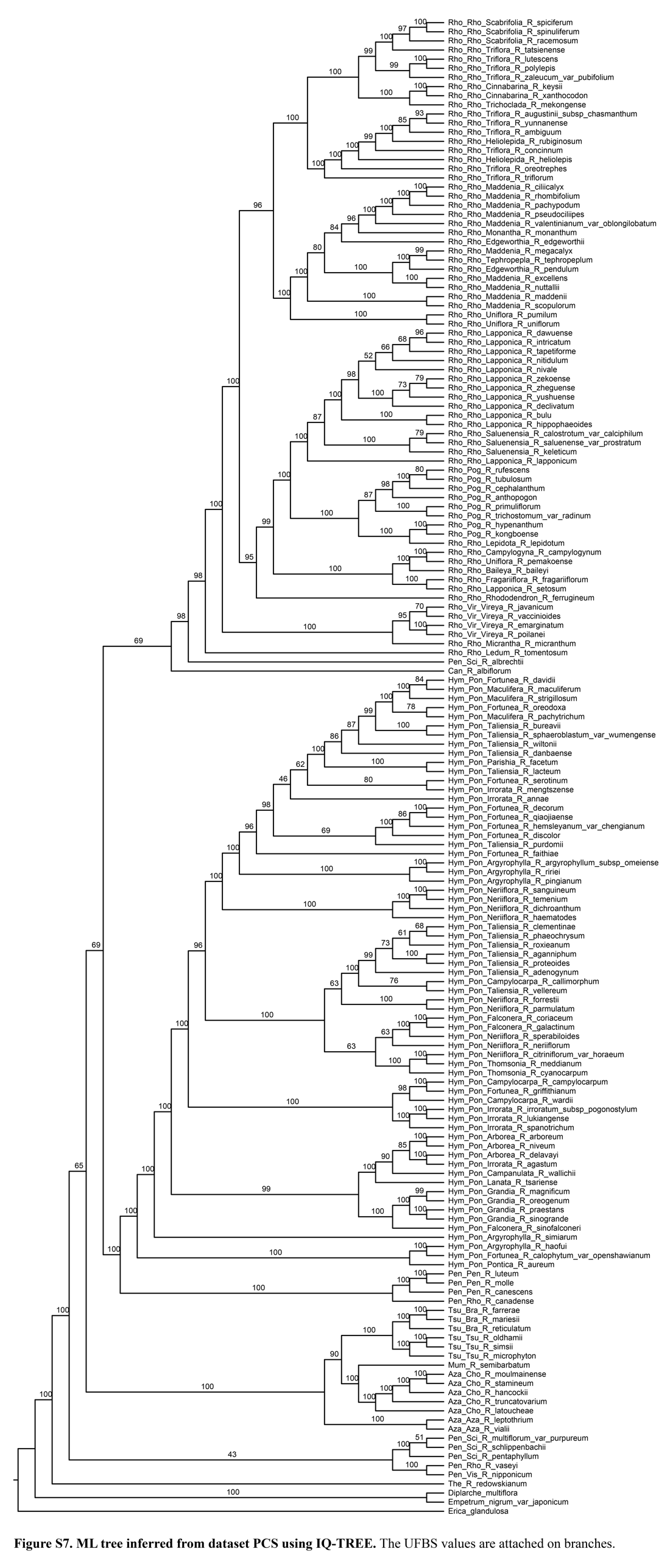

Supplement: mcac114_suppl_Supplementary_Figure_S7 [file mcac114_suppl_supplementary_figure_s7.jpeg]

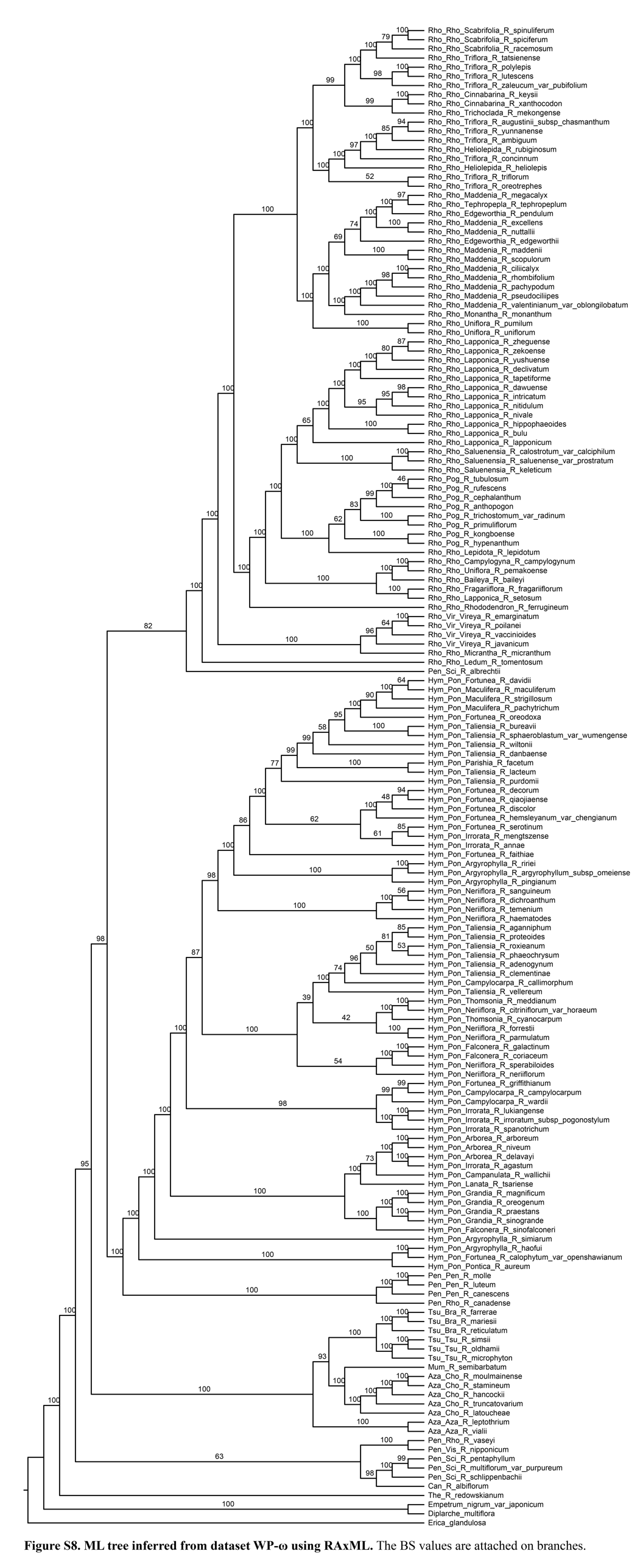

Supplement: mcac114_suppl_Supplementary_Figure_S8 [file mcac114_suppl_supplementary_figure_s8.jpeg]

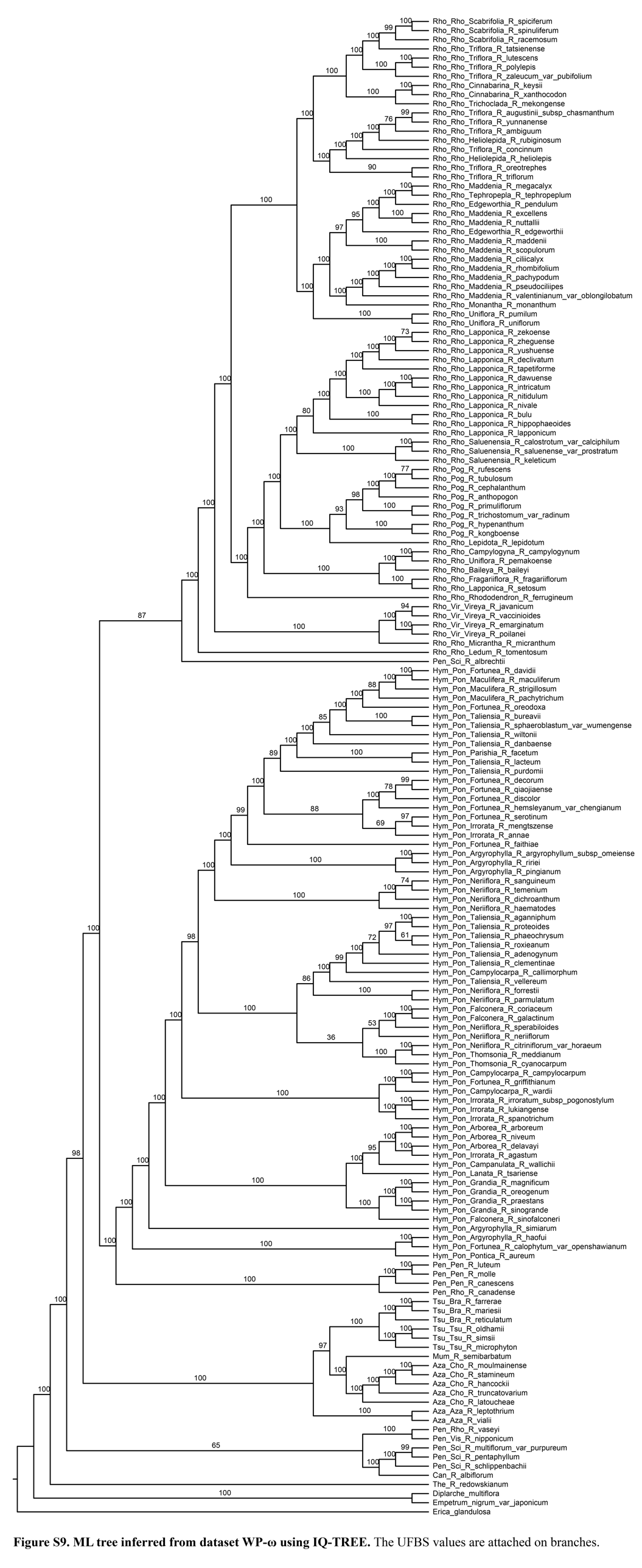

Supplement: mcac114_suppl_Supplementary_Figure_S9 [file mcac114_suppl_supplementary_figure_s9.jpeg]

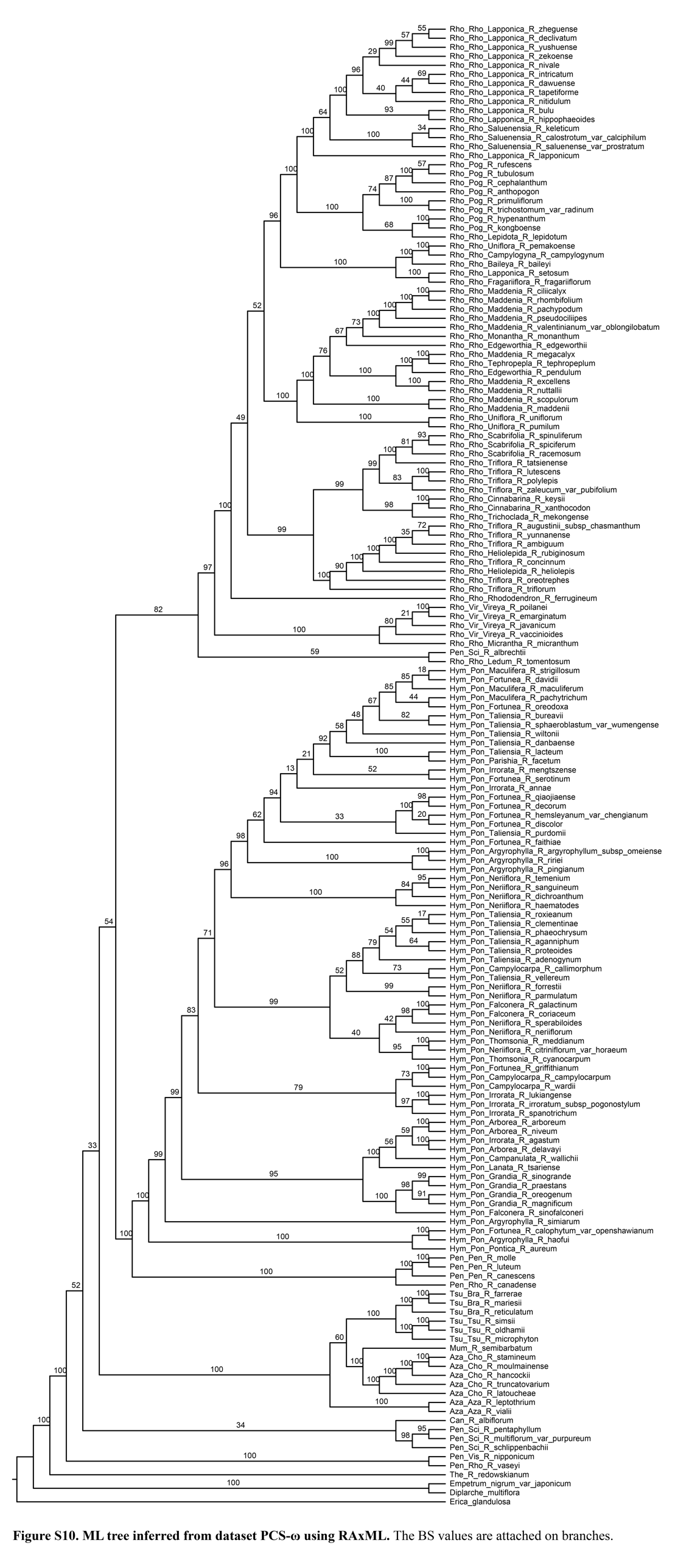

Supplement: mcac114_suppl_Supplementary_Figure_S10 [file mcac114_suppl_supplementary_figure_s10.jpeg]

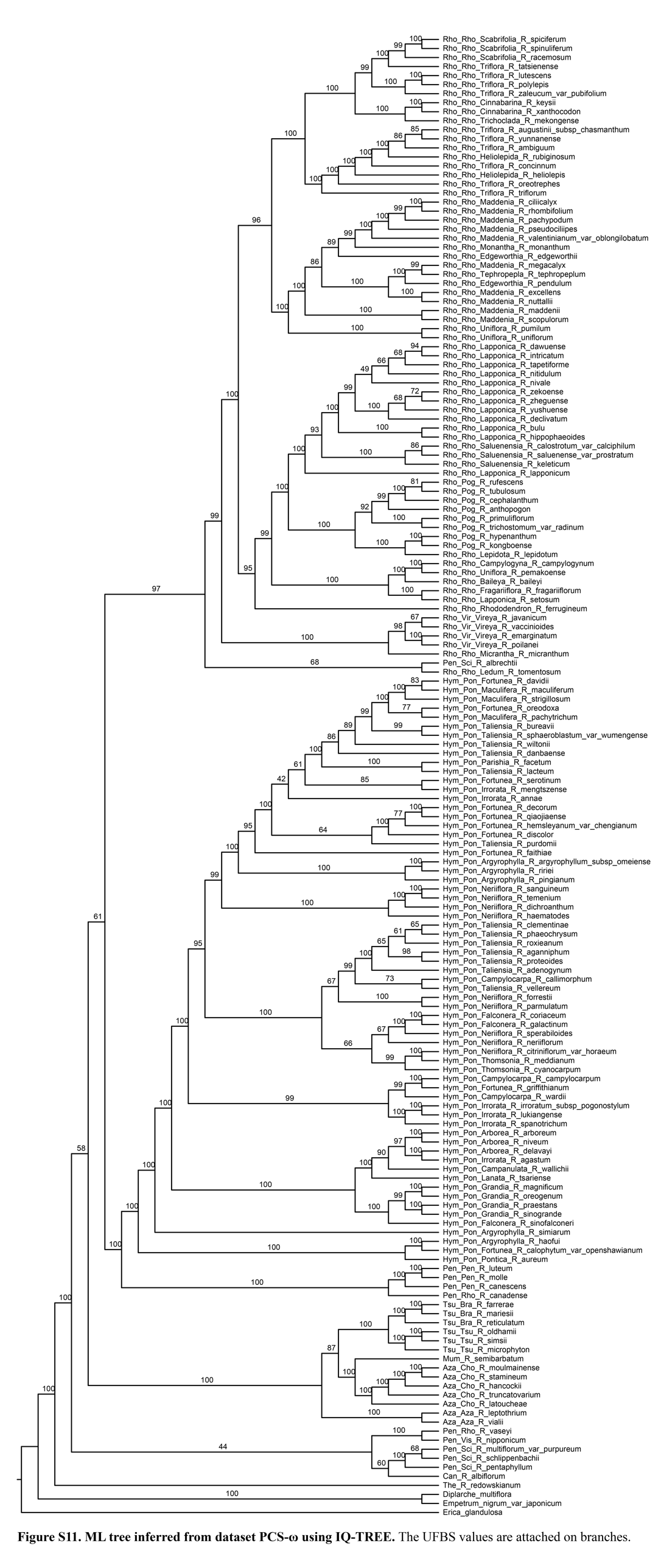

Supplement: mcac114_suppl_Supplementary_Figure_S11 [file mcac114_suppl_supplementary_figure_s11.jpeg]

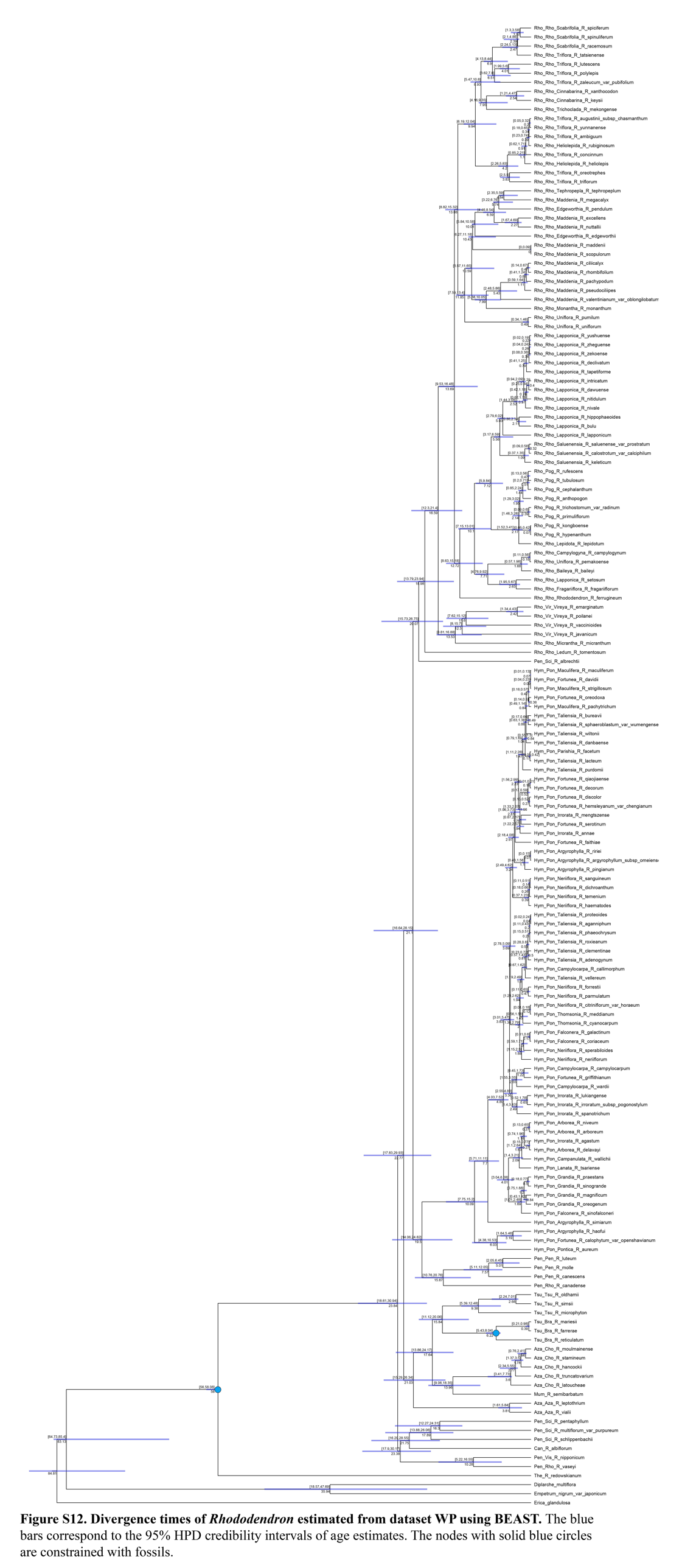

Supplement: mcac114_suppl_Supplementary_Figure_S12 [file mcac114_suppl_supplementary_figure_s12.jpeg]

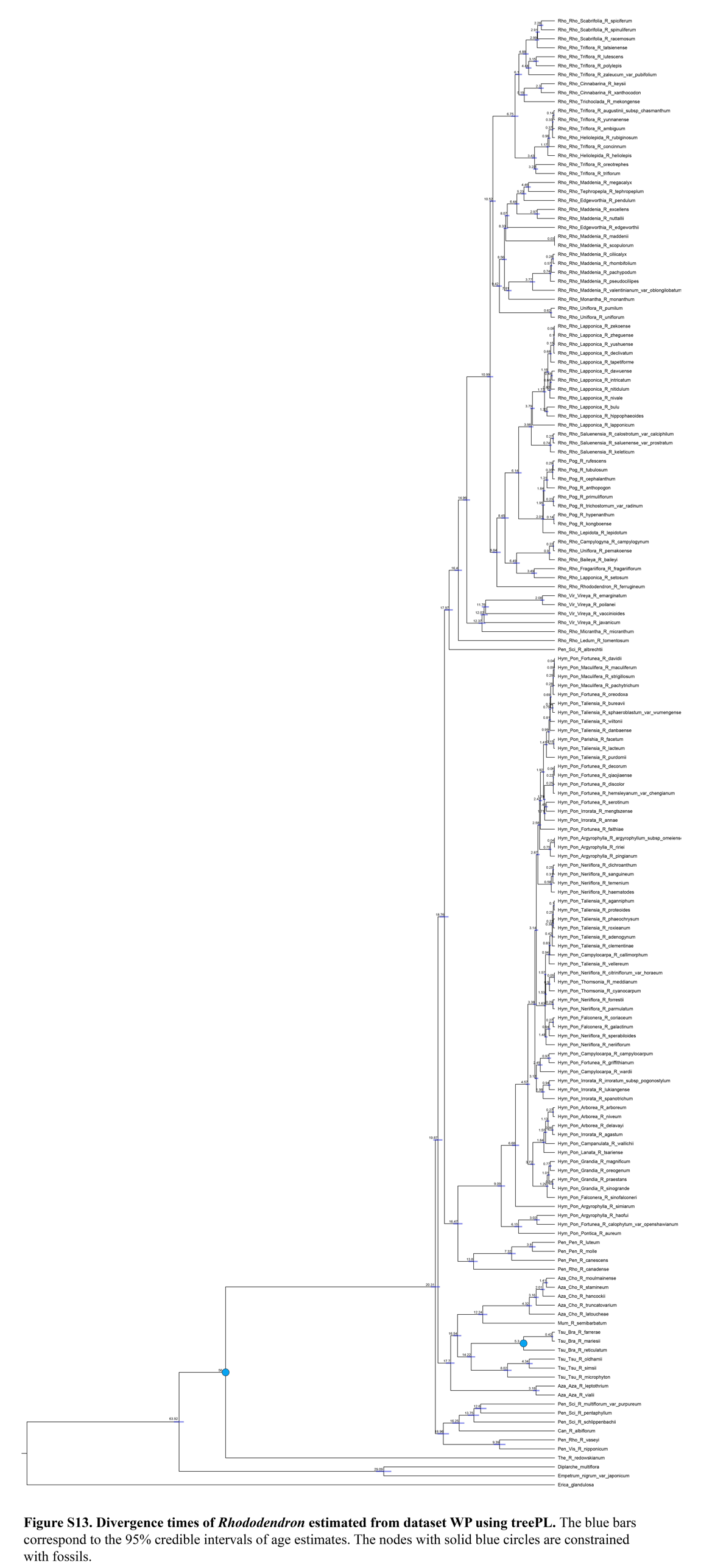

Supplement: mcac114_suppl_Supplementary_Figure_S13 [file mcac114_suppl_supplementary_figure_s13.jpeg]

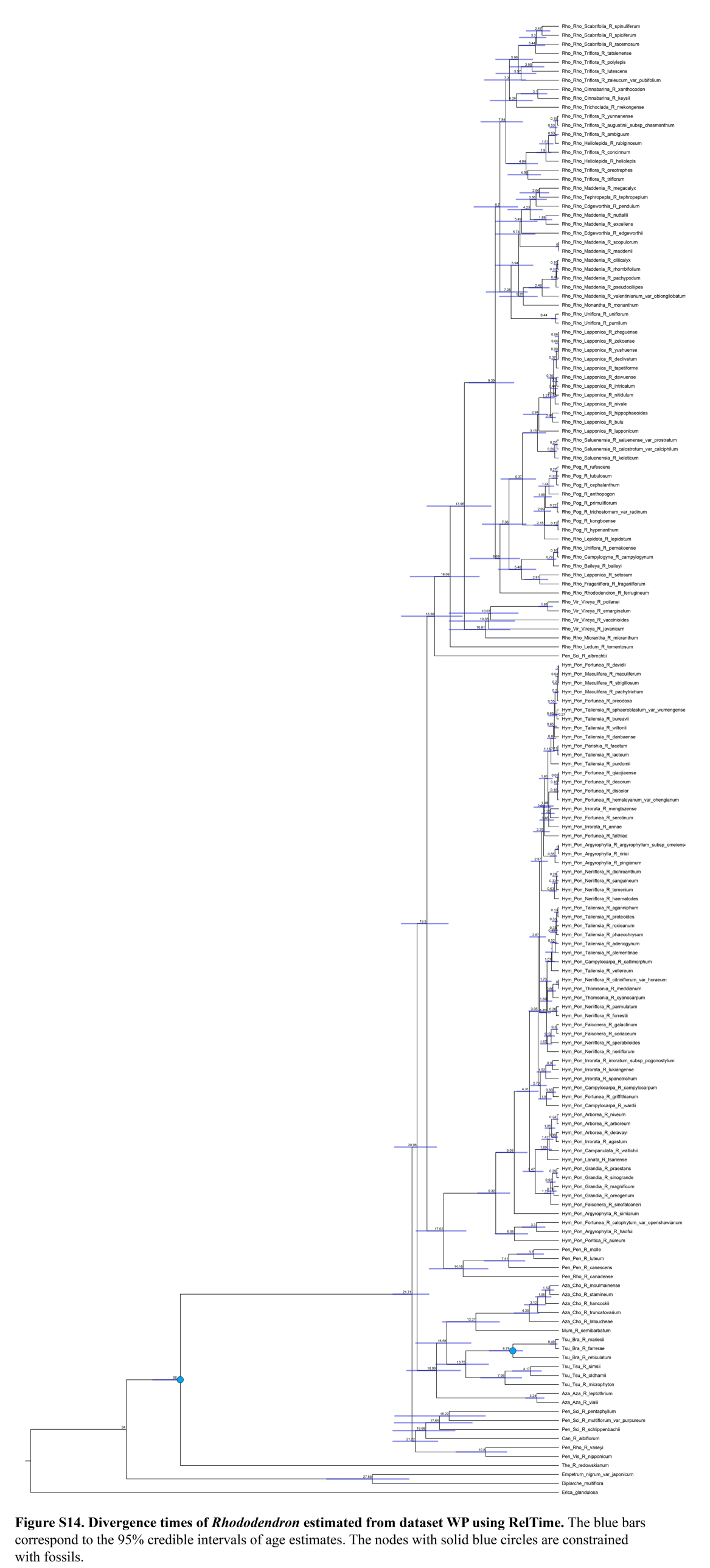

Supplement: mcac114_suppl_Supplementary_Figure_S14 [file mcac114_suppl_supplementary_figure_s14.jpeg]

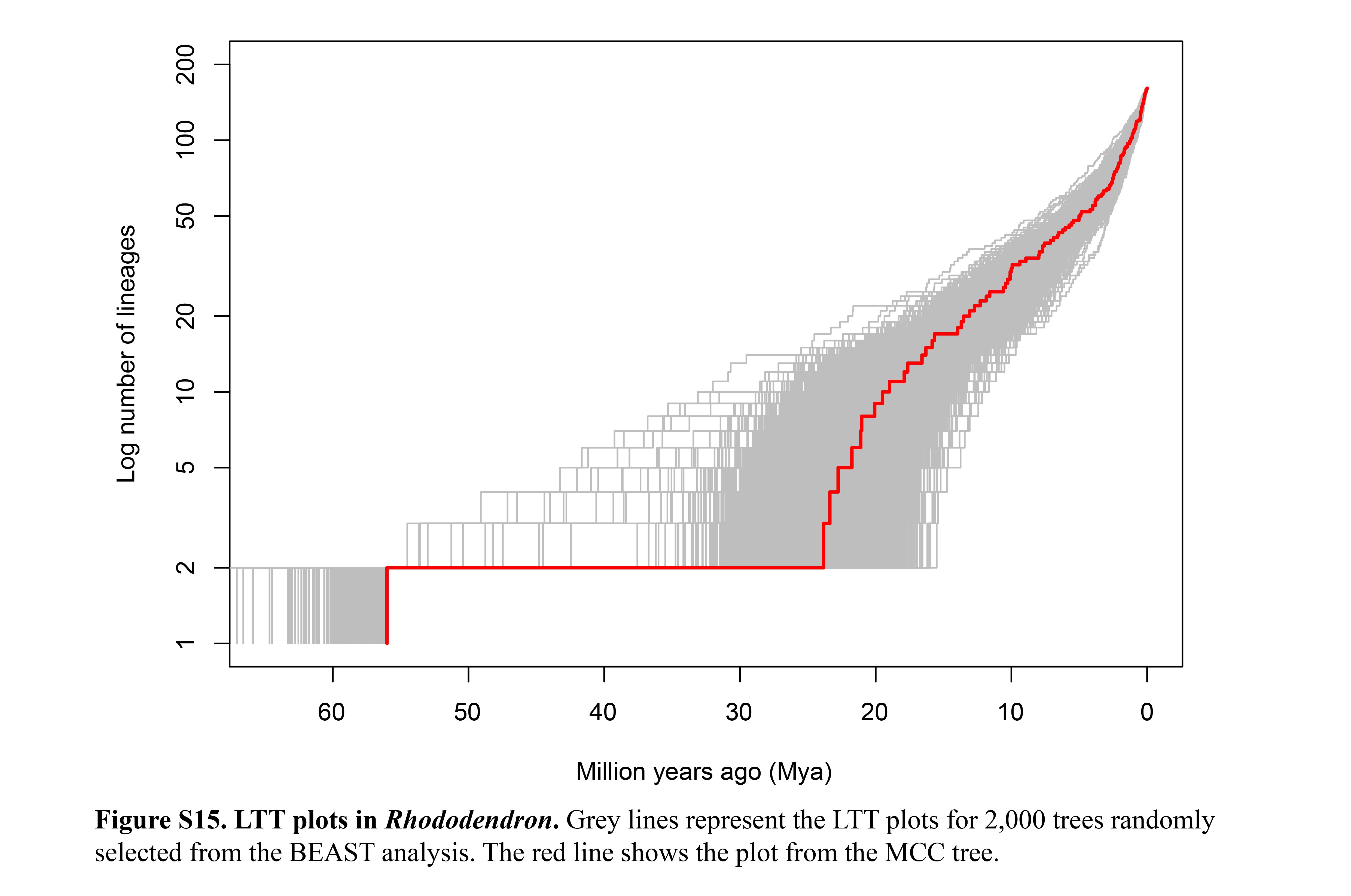

Supplement: mcac114_suppl_Supplementary_Figure_S15 [file mcac114_suppl_supplementary_figure_s15.jpeg]

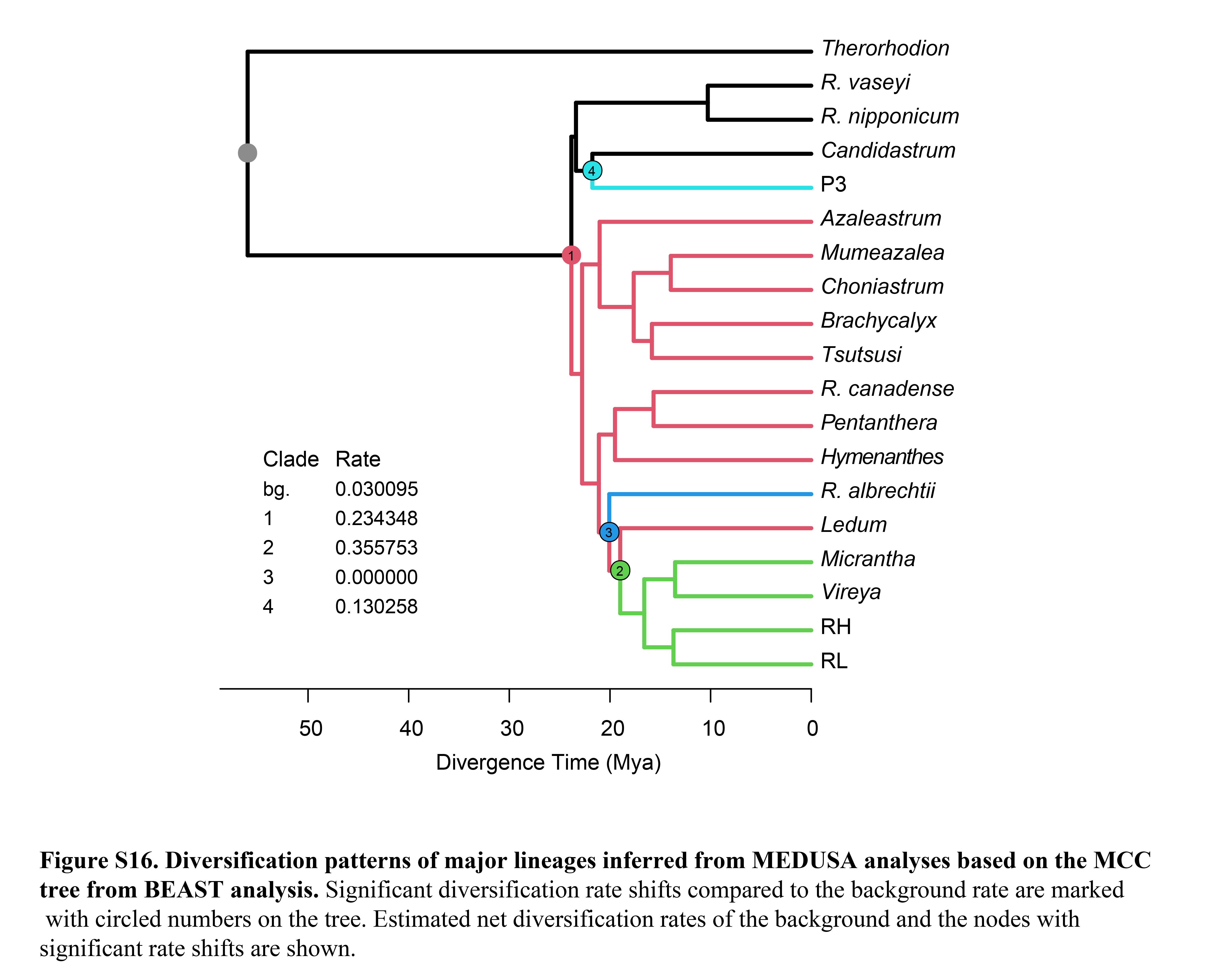

Supplement: mcac114_suppl_Supplementary_Figure_S16 [file mcac114_suppl_supplementary_figure_s16.jpeg]
